# Investigating the Acceptability and Feasibility of Three Online Interventions for Caregivers of Infants with Feeding Difficulties

**DOI:** 10.1177/00469580251375911

**Published:** 2025-10-18

**Authors:** Leanne Jackson, Ruth Drury, Giovanni Paolo Azzaro, Eduardo Coutinho, Leonardo De Pascalis, Vicky Charnock, Sian M. Davies, Clare Jones, Helen McIlroy, Sharon Remmington, Hannah Sloan, Melanie Thomas, Francine Verhoeff, Victoria Fallon

**Affiliations:** 1 University of Liverpool, UK; 2 Alder Hey Children’s NHS Foundation Trust, Liverpool, UK; 3 Maternity Voices Partnership, Liverpool Women’s Hospital, Liverpool, UK

**Keywords:** colic, milk hypersensitivity, gastroesophageal reflux, pilot projects, self-care, caregivers, maternal health

## Abstract

Colic, Gastro-Oesophageal Reflux (Disorder; GOR[D]) and Cow’s Milk Protein Allergy (CMPA) are common infantile afflictions in the first 6 months of life. These conditions are associated with high levels of infant irritability, prescription costs, and poor caregiver wellbeing. For other perinatal mental health concerns, for example, postpartum depression, peer support, music, and health education have been identified as effective interventions for nurturing caregiver wellbeing. However, these interventions have yet to be piloted in an online delivery format, among caregivers of infants diagnosed with colic, GOR(D), and CMPA. The current study aimed to determine the acceptability and feasibility of a non-clinical peer support, health education, and music intervention to caregivers of infants with colic, GOR(D), and CMPA, when compared with treatment as usual. Eligible caregivers were recruited during routine appointments with the infant feeding team at Alder Hey Children’s NHS Foundation Trust. Consenting caregivers were assigned to 1 of the 4 intervention arms. For peer support only, a WhatsApp group accompanied group sessions. Intervention weeks 1-3 involved a one-hour online group session, where skills were developed with an aim to improve management of infantile symptoms, and to nurture self-care practices. In weeks 4-6, participants were encouraged to use skills obtained from weeks 1-3, independently. In week 7, evaluative focus groups were conducted. WhatsApp group data underwent conversational analysis and evaluative focus group data underwent thematic analysis. Feasibility was not achieved due to recruitment difficulties. However, the peer support intervention was deemed acceptable by mothers and staff. Peer support participants valued the flexibility of access to support via WhatsApp with other mothers with shared life experience. Evaluative focus groups identified study strengths and limitations which will provide insight to digital health researchers seeking to develop interventional research for caregivers of infants afflicted with colic, GOR(D), and/or CMPA.

**Highlights**

● The current study aimed to establish acceptability and feasibility for three online group interventions to caregivers of infants with colic, GOR(D), and/or CMPA, when compared with treatment as usual.

● The peer support intervention was acceptable. Validating one another’s experiences and normalising infantile symptoms empowered mothers.

● Signposting caregivers to colic, GOR(D), and/or CMPA-specific parenting groups is recommended to enhance caregiver coping, self-efficacy, and emotional wellbeing.

● Overall, the study was unfeasible due to exceptionally poor uptake during recruitment.

● Evaluative focus groups identified key areas for study design revision, including placing recruitment in community health visiting services to improve participant uptake.

## Introduction

Infant colic (hereafter referred to as ‘colic’) is defined as full force crying for at least 3 hours per day, on at least 3 days per week, for 3 continuous weeks or more.^1^ Cow’s Milk Protein Allergy (CMPA) is a common food allergy in infancy which is described as a reproducible, adverse reaction to 1 or more cow’s milk protein(s).^2^ Reaction severity differs and may manifest with skin, respiratory, and/or gastrointestinal symptoms, for example, eczema, rhinitis, vomiting, or loose stools.^3^ Gastro-oesophageal reflux (GOR) is when stomach acid flows back up the oesophagus: it is a normal physiological process which can occur after eating and affects healthy individuals of all ages.^4^ When GOR causes troublesome symptoms or complications, for example, regurgitation of milk, it is referred to as GOR(D).^5^

Colic, GOR(D), and CMPA are common in the first 6 months of life^6-8^ and are associated with high levels of infant irritability and, in the cases of colic and CMPA, unsettledness.^9^ Infants with these conditions have high rates of hospital attendances and prescription costs of medication(s) and of special formula milk(s). Furthermore, colic, GOR(D), and CMPA symptoms are often overlapping or comorbid, rendering accurate diagnosis arduous and time-consuming.^10^ Lengthy treatment pathways contribute to experiences of high parental anxiety and stress; frustration, poor self-efficacy; and poor sleep quality commonly observed among caregivers of infants who are affected by these conditions.^11-13^ Caregiver wellbeing may also suffer due to the conflicting messages and inconsistent information provided by healthcare professionals regarding treatment options, for example, one healthcare practitioner may recommend a particular brand of specialist milk to aid in managing infantile symptoms, which may be then discouraged in a later appointment by a different healthcare practitioner.^14^ Since Colic, CMPA, and GOR(D) symptoms usually self-resolve, interventions have traditionally focused on promoting parental coping and infantile symptom management.

Infant irritability in public, for example, full-force crying, is perceived negatively by the general public.^15^ Additionally, new parents also frequently report concerns that one’s inability to settle their infant when they are distressed may depict them as an inadequate mother.^14^ Such concerns are especially pronounced among babies who have colic, reflux, and CMPA.^1^ This stigmatisation can result in help-seeking avoidance among caregivers for their baby’s condition, due to fears of receiving negative judgement from others.^16^ This manifests in an overreliance on emergency services, once infantile symptoms have become especially acute.^17^ Delivering caregiver support online offers an opportune solution.^18,19^ Specifically, peer support,^20^ health education,^21^ and music interventions^22^ have been significantly associated with improved maternal wellbeing and perceived infant settledness. Likewise, WhatsApp groups have proven fruitful when used in postnatal interventions.^23^ To the authors’ knowledge, however, there have been no attempts to investigate the respective feasibility and acceptability of peer support, health education, and music interventions in a single study. Additionally, no previous research has investigated the potential benefit of these respective interventions to a population of caregivers with unsettled infants due to colic, GOR(D), and/or CMPA, when delivered in an online group format.

An empty trial refers to a study which contains exceptionally poor intervention: uptake and/or high attrition, which has hindered ability to evaluate intervention arms.^24^ Reporting unfeasible and unsuccessful research holds as much academic merit as successful trials.^25^ Certainly, transparent reporting is the cornerstone of sound scientific reporting.^26^ This argument, too, has been corroborated by the Strengthening The Reporting of Observational studies in Epidemiology (STROBE) guidelines for experimental trials.^26^ For example, reporting on reasons for drop-out at each stage of study conduct allows important comparisons to be made between non-participants and participants, which can benefit future research endeavours.^27^

Manuscripts yielding significant findings are more likely to be accepted for publication, are published more quickly, and in higher impact journals when compared with research reporting unconventional or null findings.^28^ Issues pertaining to publication bias in this manner have diminished confidence in conclusions drawn from interventional research, due to overstated and inflated positive reporting.^29^ Empty trials can offer important insights when designing interventions, by reflecting on and avoiding unsuccessful practices. To illustrate this point, common barriers to participant retention in feasibility research include cumbersome time commitments (eg, having many follow-up sessions) and recruiting from sensitive populations (eg, caregivers of infants experiencing colic).^30^ The current study is considered an empty trial due to the low final sample size of seven participants, of a desired final sample of forty participants.

### The Current Study

The current study aimed to determine the acceptability and feasibility of an online, group: peer support, health education, and music intervention to caregivers of unsettled infants with colic, GOR(D), and/or CMPA, when compared with treatment as usual. A mixed-methods approach was adopted to address this overarching aim. Firstly, psychometric measures distributed pre- and post- intervention were compared across respective intervention arms. It was hypothesised that, when compared with treatment as usual, caregivers assigned to one of the three intervention arms would score higher in parental confidence, satisfaction with healthcare professional support, and would score lower on measures of postnatal depression and anxiety when compared with treatment as usual. A significant difference between respective intervention arms on distributed measures was expected. Post-intervention focus groups were also conducted with participants and with staff, separately, to evaluate perceived benefit of the respective intervention arms in greater depth. Finally, a conversation analysis was conducted on WhatsApp group data, which spanned the peer support intervention arm, only.

## Methods

The current study was reported in line with CONSORT pilot and feasibility trial guidelines.^31^

### Ethical Statement

Research Ethics Committee (REC) and Health Research Authority (HRA) ethics approvals were attained on 21 Oct 2021 (ID: 21/NW/0258). Sponsor approval from The University of Liverpool was obtained on 25 November 2021 (Sponsor Ref: UoL001641).

### Study Design

The present study was pre-registered through the International Standard Randomised Controlled Trial Number (ISRCTN) registry on 06 August 2021 (ID: ISRCTN15349263). The full study protocol can be found through this pre-registration. The current pilot study aimed to investigate the feasibility and acceptability of an online group peer support, health education, and music intervention for caregivers of infants affected by colic, reflux, and GOR(D).

In the current study, the largest risk of harm was psychological distress. Measures were taken throughout the design and implementation of the study to protect caregiver wellbeing. For example, a distress protocol was developed for the unfortunate event that a participant became upset during the study, which can be made available on reasonable request to the corresponding author. All group sessions and the evaluative focus groups were attended by two members of the research team for in the event that the distress protocol needed to be implemented. Consent was taken electronically and verbally during the intervention and focus group, respectively, to respect consent as a continuous process. Participants were reminded in the information sheet and at the start of each session of their ability to withdraw from the study at any point and without needing to give a reason.

The peer support group carried additional risk of harm when compared with music and health education. Specifically, setting up the WhatsApp group meant sharing the participant’s mobile number and profile picture with other peer support intervention participants. Caregivers were made aware of this feature of the study before deciding to take part, and were given instructions on how to mute notifications, block contacts, and remove their profile picture icon if they felt uncomfortable at any point. Participants were reminded that they could interact with the other members as much or as little as they liked throughout the peer support intervention. The first group session of the peer support intervention laid ground rules for appropriate behaviour in the WhatsApp group, for example, not creating splinter groups, not discussing medical advice in the group, keeping anything discussed in the WhatsApp group completely confidential etc. Powerpoint slides for this session can be made available on reasonable request to the corresponding author.

The WhatsApp group was monitored by a member of the research team to ensure that ground rules were being followed [SD]. Research participants were made aware of this feature of the study before deciding whether to take part. Participants were also informed that the WhatsApp group chat would be analysed and written up into a report. Participants were informed in the information sheet and first group session that if they preferred for any message(s) not to be included in final analysis, that they should either (i) put a padlock emoji on the message, or, (ii) include a note in the message asking for their message(s) to be excluded from final analysis. See pre-registration for the full study protocol, which provides extensive information on measures emplaced to protect participants from harm throughout study design and conduct.

### Participants

Recruitment was opportunistic: mothers who had been referred to Alder Hey Children’s NHS Foundation Trust (Hereafter referred to as ‘Alder Hey’) with their unsettled infant, due to infant feeding difficulties relating to Colic, GOR(D), and/or CMPA, were identified by clinicians employed at Alder Hey. All caregivers who met the following eligibility criteria were approached by their clinician during routine appointments: over the age of 18, and no previous or current clinical diagnosis of a serious mental health condition, that is, bipolar disorder, schizophrenia, and/or psychosis (confirmed via self-report on initial contact [LJ], described below). Infants needed to be younger than six months old at time of referral, born full-term (ie, >34 weeks' gestation), without known comorbidities or evidence of faltering growth^34^ for their caregiver to be eligible to participate.

If interest was expressed on clinician approach, the parent was given an information pack containing a study advertisement, information sheet, and expression of interest form. Eligible caregivers completed and returned the expression of interest form. Contact details were securely transferred to LJ and an initial call was made after approximately 48 hours. During the initial call LJ confirmed caregiver eligibility, provided information on the intervention arm to which they had been assigned, and answered any questions about the study. An email was then sent to the participant, containing: an electronic consent form, an active Qualtrics survey link to complete an initial baseline survey (approx. 5-10 min completion time), and details of the first group Zoom intervention session.

### Group Allocation and Intervention Details

A pragmatic sample of 40 participants was sought.^32^ Treatment as usual ran first due to lack of prior preparation required to set up the intervention arm. Clinician capacity meant that six weeks’ notice was required to block out sufficient time to run the health education intervention alongside their usual caseloads. Therefore, the health education intervention arm ran third, and the peer support intervention arm ran second due to lack of pre-preparations needed to run this intervention arm. Music intervention ran last due to capacity of the self-employed harpist, who facilitated scheduled group intervention sessions.

Each intervention arm ran either i) once 10 participants had been recruited to the arm, or, failing this target being met, ii) After one month of continuous recruitment efforts. Inability to recruit 10 participants to an intervention arm within one month of continuous efforts was deemed as the arm being unfeasible for scaling up to a full intervention. Principally, this decision was made to recognise the self-resolving nature of infantile symptoms for these conditions, that is, to maximise intervention benefit to parents who had been screened into the study earlier in the recruitment bracket.

Treatment as usual ran in February 2021, after 1 month of continuous recruitment efforts in January 2021. Two women participated in treatment as usual, of 14 contacted (14% uptake). One father took part in the treatment as usual focus group, only, on participant request. Peer support ran in March 2021, after 1 month of continued recruitment efforts in February 2021. Three women were recruited to peer support, of 10 contacted (30% uptake). One participant was recruited to music, of 20 contacted (.05% uptake). The music intervention ran in March 2021 after one month of continuous recruitment efforts in February 2021. However, the singular participant chose to withdraw after the first online session. This participant withdrew due to discomfort taking part in the intervention arm alone, due to its advertisement as a group intervention.

No participants were recruited to health education after one month of continuous recruitment efforts in April 2021: eight women had been contacted (0% uptake). Non-participating individuals either i) did not respond to three attempts at contact, spaced 24 hours apart, or ii) hung up on two consecutive contact attempts, spaced 24 hours apart. See Table 1 for participant demographic information.

**Table 1.** Participant Characteristics and Pseudonym Assignment.

Intervention arm	Parent pseudonym	Infant pseudonym	Number of children (including youngest)	Parental age	Occupation	Highest level of education	Marital status	Youngest infant age/months	Infant feeding method
Treatment as usual	Hannah	Willow	2	NR*	NR*	NR*	NR*	20	Formula feeding
Treatment as usual	Freida+	Kimberly	1	32	Associate professional occupation	College	Living with partner	2	Combination feeding
Treatment as usual^^	Terry+	As Frieda	As Frieda	28	Managers, directors, and senior officials	Undergraduate Degree	Living with partner	As Frieda	As Freida
Peer support	Barbara	William	1	34	Professional occupations	Master’s Degree	Married	4	Combination feeding
Peer support	Silvia	George	1	28	Managers, directors, and senior officials	Master’s Degree	Living with partner	2	Formula feeding
Peer support	Madison	Billie	2	29	Professional occupations	Undergraduate Degree	Single	5	Formula feeding
Music	Alanta	NR*	NR*	NR*	NR*	NR*	NR*	NR*	NR*

NR*: Field received no response. Please note that occupation categories and education categories were taken from the UK Government (2020) and (2021), respectively.

+ Terry and Frieda are cohabiting partners.

^^ This participant only took part in the focus group, on Frieda’s request.

At the rate of recruitment recorded in the current study, that is, between one and three participants being screened to an intervention arm per month, each intervention arm would run after approximately four to ten months of continuous recruitment efforts. This is problematic because a parent recruited in month one would likely have very different support needs than a parent recruited in month 10, making evaluation of intervention benefit impossible. More problematic than this, parents recruited earlier in the recruitment bracket may have infants whose symptomsself-resolved by the time that the intervention runs. This may have caused undue distress by having been recruited to a study which they were then unable to benefit from, due to reasons outside of their own control.

Where it has been feasible to do so, intervention arm design has been standardised. All intervention arms spanned seven weeks. For weeks one through three, each participant was invited to attend a one-hour online session. Each session was split into two sections: in the first 40 minutes the delivering member of staff taught mothers techniques and/or skills to use with their unsettled infant (the content of which was adapted for the intervention arm). The remaining 20 minutes of each online session was used as an opportunity for any questions and/or informal socialising among the attendees. In intervention weeks four through six, parents were told to use the skills and/or techniques acquired during the group sessions as much or as little as they would like, in their own time.

To further standardise our approach, each online group session took place on a Monday morning. On the Friday morning of the same intervention week, participants were sent an interim questionnaire to assess how useful they found the intervention content for that week, and how much they had engaged with it. Questions were tailored for each intervention arm, for example, ‘Did you find [using songs of kin e.g., lullabies/using the WhatsApp group] helpful this week, when your baby was unsettled?’. All participants were also sent the same psychometric measures at baseline and post-intervention.

All participants took part in an evaluative focus group for the pilot study, which took place in week seven, in the Monday morning slot whereby the group online sessions had previously been taking place. In week six, participants were sent the baseline survey from week zero to complete again so that the research team could determine change over time. After completing this post-survey, participants were redirected to a downloadable debrief sheet on Qualtrics and then were asked if they would be interested in taking part in an audio recorded focus group to share their experiences of taking part in the study. If the participant selected ‘yes’, they were re-directed to a separate Qualtrics survey where they were presented with an information sheet for the focus group, and a consent form to sign. Participants who signed the focus group online consent form were then sent details of the scheduled focus group. Please see Appendices A-D for a more detailed breakdown of each intervention arm’s design. PowerPoint slides for each group session can be made available on reasonable request to the corresponding author, and details of intervention arm delivery are summarised below.

#### Treatment as Usual

All participants continued to receive their usual care at Alder Hey. Participants in all intervention arms received treatment as usual.

#### Health Education

Infant feeding specialists employed by Alder Hey, led organised group sessions (CJ, HM). Sessions aimed to normalise infantile symptoms through education and the provision of tailored advice and guidance on symptom management. Group sessions covered the following topics: understanding colic, GOR(D), and CMPA; symptom management; and both infant development and weaning behaviour. Group sessions were 40-minutes in length followed by 20-minutes of Q&A’s.

#### Music

An arts coordinator and arts psychotherapist employed by Alder Hey [SR, MT], and an academic researcher employed by The University of Liverpool, who specialises in the psychological study of music [EC], led organised group sessions. This intervention aimed to educate caregivers on the use of songs of kin, for example, lullabies, to manage infantile symptoms and to improve caregiver mood and confidence. Group sessions covered the following topics: the benefits of Infant-Directed Singing (IDS^36^) developing parental confidence using IDS and addressing barriers and/or anxieties for its use. Group sessions were 40-minutes in length followed by 20-minutes of Q&A’s.

#### Peer Support

Academic researchers employed by The University of Liverpool, who specialise in perinatal mental health and infant development research (LJ, SD, VF) and a Maternal Voices Partnership (MVP) representative (HS) led organised group sessions. The aim of this intervention arm was to improve caregiver mood and confidence via social cohesion and normalising infantile symptoms by sharing lived experiences. Topics covered in group sessions included: what peer support is and why it is beneficial; ground rules for using the WhatsApp group; success stories from parents with previous experience of caring for an infant with colic, GOR(D), and CMPA; managing challenges; and use of self-care strategies.

### Pre-and Post-assessment Questionnaires

The following were administered at baseline and in week six of each intervention arm, including treatment as usual, to assess change over time:

- Infant feeding method was assessed using a validated 11-point Likert Scale with percentage response options varying from 100% formula fed to 100% breastfed over the past 48-hour period^33^
- *Perceived Maternal Parenting Self-Efficacy (PMPSE) tool*^34^: a 20-item self-report questionnaire to assess perceived parenting self-efficacy with four subscales reflecting: infant caretaking, evoking behaviour(s), reading behaviour(s) or signalling, and situational beliefs. Response options included: *‘strongly disagree’, ‘disagree’, ‘agree’*, and *‘strongly agree’*. Higher scores reflected higher parenting self-efficacy.
- *Edinburgh Postnatal Depression Scale* (EPDS^35^): a 10-item self-report questionnaire to assess postpartum depressive symptoms. Higher scores reflected higher levels of depression.
- *Postpartum Specific Anxiety Scale* (PSAS^36^): a 16-item self-report questionnaire to assess postpartum-specific anxiety symptoms. The questionnaire contained four sub-scales: psychosocial adjustment to motherhood anxieties, practical infant care anxieties, maternal competence and attachment anxieties, and infant safety and welfare anxieties. The PSAS is measured on a four-point Likert scale with response options ranging from ‘0 Not at all’ to ‘3’ Almost Always’. Higher scores on the PSAS reflected higher levels of anxiety.
- *Short Assessment of Patient Satisfaction* (SAPS^37^): a seven-item self-report questionnaire to assess perceived satisfaction with healthcare professional support. Response options included, *‘very satisfied’, ‘satisfied’, ‘Neither satisfied nor dissatisfied’, ‘Dissatisfied’*, and, *‘Very dissatisfied’*. Higher scores on this scale reflected higher perceived satisfaction with healthcare professional support.

### Procedure

After providing consent, the following demographic information was recorded in the baseline survey: maternal age, infant age, infant feeding method, occupational status, highest level of education, marital status, and parity. Each intervention arm ran for seven weeks. For peer support, music, and health education, weeks one through three consisted of attending a weekly, one-hour online group session. For which, session information was distributed via email (LJ). In weeks four through six, participants were asked to implement the skills and techniques acquired in the group sessions, independently. Interim questionnaires were administered each Friday in intervention weeks two through five, to assess frequency and quality of engagement, and perceived helpfulness of the intervention arm (See Appendices A-D for treatment as usual, peer support, music intervention, and health intervention protocols, respectively).

Evaluative focus groups took place in week seven for all intervention arms, and for delivering members of staff to explore acceptability and feasibility in greater depth (See Appendix E and F for intervention and staff focus group topic guides, respectively). A WhatsApp group accompanied the peer support arm, only, with participant consent. Participants were informed that they could interact with one another as much or as little as they liked outside scheduled intervention sessions, including face-to-face meetups. The WhatsApp group was monitored, and conversational prompts were periodically posted to facilitate conversation (SD).

### Analysis

#### Pre-, Post- and Interim Assessment Measures

No participants completed pre- or post-intervention measures. Neither did any participants complete interim assessment measures. Coupled with exceptionally poor levels of recruitment, quantitative examination of pre-, post-, and interim assessment measures was not possible.

#### Conversation Analysis

All consented exchanges from the peer support WhatsApp group were exported to NVivo 12, and a conversational analysis was conducted^38^ [GPA, LJ]. Here, talk-in interactions were assessed, for example, turn-taking.^39^ The structural organising rules of talk can be detected by analysing sequences of naturally occurring conversation, where a bidirectional relationship exists between social interaction and context.^40^ Analysis was inductive and consultative.^41^

#### Reflexive Thematic Analysis of Focus Groups

Topic guides were reviewed by all members of the research team, who collectively possessed expertise in clinical care, perinatal and qualitative research methods, and lived experiences of parenting an infant with colic, GOR(D), and/or CMPA. The intervention topic guide covered: motivations for participation, appropriateness and experience of recruitment, satisfaction with the intervention arm (mode, frequency and type of delivery, benefit; Appendix E). The Treatment as usual topic guide fed into a service AUDIT for Alder Hey, and is thus, not presented in the current paper. The staff topic guide covered: motivation for working on the current project; satisfaction with involvement in the study; views and experiences of study design, recruitment, and session delivery (Appendix F).

Peer support and staff focus groups ran in March and June 2021, respectively. Interviews lasted between 60-91 minutes (Mean = 73 minutes). The peer support focus group was conducted via video conference calling software and the staff focus group was conducted face-to-face at The University of Liverpool. All participants were debriefed within 24 hours of the focus group concluding. The peer support focus group was led by LJ and facilitated by SD, while the staff focus group was led by an independent researcher who was otherwise unaffiliated with the current study, and blind to its aims. Focus groups were audio recorded using a Dictaphone and transcribed verbatim. Transcription and analysis were supported by NVivo 12 (RD, LJ). Researchers used reflexive thematic analysis,^42^ involving familiarisation with the transcripts, generation of initial codes, identification, review, and defining themes, and report writing (RD, LJ). All authors reviewed and refined identified themes, following an inductive and consultative approach,^41^ to derive the final thematic structure. See supplementary documentation for author reflexivity statement.

## Integrated Results and Discussion

The current study aimed to investigate the feasibility and acceptability of online group peer support, health education, and music interventions to caregivers of infant’s referred to Alder Hey, due to infant feeding difficulties relating to colic, GOR(D), and/or CMPA.

### Peer Support: Conversation Analysis

**Figure 1.**
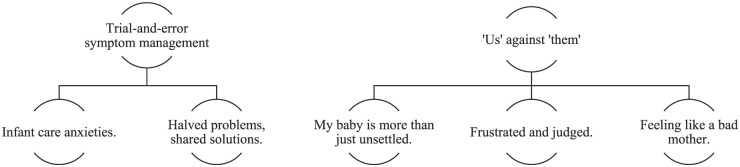
Conversation analysis thematic structure.

Conversation analysis identified 2 major themes, ‘*Trial-and-error symptom management*’ and, *‘Us’ against ‘them’*. Five sub-themes were identified: ‘*Infant care anxieties’, ‘Halved problems, shared solutions’* (trial-and-error symptom management), *‘My baby is more than just unsettled’, ‘Frustrated and judged’*, and *‘Feeling like a bad mother’* (*‘Us’* against *‘Them’*). Figure 1 provides a diagrammatic overview of the final thematic structure.

*‘Trial-and-error symptom management’* was characterised by the sharing, validation, and normalisation of one another’s difficulties with unsettledness management. The second major theme, *‘’Us’* against *‘them’*, captured the use of humour, rapport, and group membership to alleviate otherwise distressing and shame-inducing experiences. Rapport built between WhatsApp group members countered the negative effects of perceived negative judgement from others regarding their infant’s condition (See Appendix G for supporting quotes). Elevated anxiety was attributed to upward comparisons made with caregivers of infants unafflicted by Colic, GOR(D) and/or CMPA. For the multiparous participant, upward comparisons were also made with their older, unaffected child. This may be explained by self-discrepancy theory: when one believes themselves to be failing self-imposed expectations, feelings of inadequacy, guilt, and shame ensue.^43^ Ideal-actual comparisons can lead to the onset and maintenance of other psychiatric symptoms, such as postpartum anxiety and depression.^44^ This is especially pronounced among mothers who have infants with colic, GOR(D), and/or CMPA.^11-13^

Symptom management anxieties were intensified by healthcare professional support when perceived to be unhelpful, dismissive, and inconsistent. In other domains of maternity care, such as infant feeding, receiving care which was not mother-centered resulted in poorer health outcomes and dissatisfaction with care, while satisfaction protected maternal emotional wellbeing and optimised infant health outcomes.^45^ Current findings reaffirm the need for holistic, mother-centered care which considers maternal emotional wellbeing and autonomy as key intermediaries of maternal and infant wellbeing outcomes.^46^ Healthcare practitioners are in an influential position to protect the *‘good mother’* by closing the ideal-actual discrepancy. To do this, proactive normalisation of infantile symptoms, dispelling myths, and tailoring advice to maternal needs is necessary.^45^

Parenting guilt is unfortunately common.^45^ Under intensive motherhood theory, infant unsettledness deviates from the *‘good infant’* prototype and predisposes new parents to feelings of inadequacy.^47^ In the current study, participants avoided parenting groups due to fears of being judged by other mothers: this is characteristic of shame.^48^ Attending parenting groups with other caregivers who had shared life experiences renewed one’s ability to enjoy quality time with their unsettled infant, in public. Heightened anxiety was also common, for which the WhatsApp group acted as a platform to celebrate infant development milestones beyond their afflictions.^49^ In this regard, the WhatsApp group acted as a safe haven for participants, which nurtured the *‘good mother’* identity.^47^ This was achieved by neutralising negative judgement received from outsiders with validating and empowering assurances.^48,49^

For the peer support intervention arm, online group sessions in week two and three focused on normalising infantile symptoms, sharing lived experiences; and discussing self-care techniques, respectively (See Appendix B for more details). These sessions were particularly well received by mothers in this intervention arm, and facilitated self-acceptance, self-compassion, confidence, and perseverance as self-reported in the evaluative focus group (See Appendix G and H for supporting illustrative quotations). Self-efficacy is conceptualised as one’s belief in their ability to carry out an intention, successfully.^50^ In health research, self-efficacy predicts health status and disease prevention.^51^ The current study finds peer support to offer cost-efficient benefit to mothers of infants with colic, GOR(D), and/or CMPA, extending previous work conducted with childbearing populations.^52^ Signposting to local peer support services and to specialized helplines, for example, Cry-sis,^53^ during routine appointments, is thus recommended. Signposting to population-specific self-help resources and support groups during routine healthcare appointments may also alleviate pressure on primary healthcare services.^54,55^

### Evaluative Focus Groups, Staff, and Peer Support

See Table 2 for a thematic summary of study strengths and weaknesses. For supporting illustrative quotes and interpretations for identified themes, see Appendix H.

**Table 2.** Thematic Summary of Study Strengths And Weaknesses.

	Major theme	Sub-theme
Study strengths	Effective study design and delivery	1. A personal touch 2. Accessibility and instilled confidence
Study weaknesses	Issues with recruitment and delivery	1. Delayed point of entry 2. A mismatch of the formal and informal 3. Hybrid delivery and condensed sessions

While peer support was an acceptable intervention, its feasibility was not established due to poor participant uptake. Thematic analysis identified two overarching themes: effective study design and delivery, and issues with recruitment and delivery. In the current study, any parent who visited the infant feeding team at Alder Hey Children’s Hospital due to symptoms of colic, reflux, and/or CMPA and who met eligibility criteria, were invited to take part in the current study by their clinician. However, uptake to the current study was exceptionally poor (treatment as usual uptake: 14%, peer support uptake: 30%, music uptake: .05%, health education uptake: 0%). This resulted in overall study uptake of 10% from those who were contacted for participation. Non-participating individuals either i) did not respond to three attempts at contact, spaced 24 hours apart, or ii) hung up on two consecutive contact attempts, spaced 24 hours apart. During the treatment as usual and peer support evaluative focus groups, participants spontaneously mentioned that on approaching Alder Hey Children’s Hospital for their child’s unsettledness, they had already exhausted all community-based sources of support, for example, General Practitioner, Health Visitor.

Participants went on to express that by the time they were contacted about the current study, their infant’s symptoms had either started to subside, or they had been in a mentally exhausted position after many signposting attempts to gain support for their infant’s condition. For those interviewed, this was a pressing barrier for taking part in the current study. Delivering members of the research team agreed with the participant’s reflections. Establishing community-based recruitment, for example, through health visiting services and parenting support groups, would allow eligible caregivers to be identified at a point where level of need is greater,^54^ and might subsequently reduce overreliance on emergency services.^55^

Regarding study strengths, personalised recruitment calls with direct contact from the delivering research team facilitated intervention uptake. This echoes previous research which also demonstrated the benefits of personalised recruitment.^56^ Online intervention delivery further improved intervention accessibility: strengthening evidence for the use of videoconferencing software in peer support interventions.^57^

However, in the peer support evaluative focus group, suggestions were made to deliver the intervention arms in a hybrid format, opposed to current delivery which was entirely online. For the peer support arm, mothers reported having liked to have had the opportunity to meet face-to-face for an icebreaker activity, for example, coffee and biscuit morning, to acquaint themselves with one another. This was perceived as being particularly important given the deeply personal intervention topic. After this icebreaker activity, mothers reported having been happy with all remaining sessions being held online, as they were able to meet up as much or as little as they liked outside of these sessions.

Patient perspectives on post-COVID-19 maternity service reconfiguration echoes these findings, with perinatal women consistently expressing a desire for elements of in-person interactions.^58^ Hybrid intervention delivery with an in-person icebreaker component is a previously undocumented, novel finding. Based on these findings, the research team propose that researchers developing perinatal group interventions in future incorporate an informal icebreaking session before commencing with intervention delivery, to give participants the opportunity to start building rapport. Condensing intervention sessions from 60 minutes to 45-minutes was also suggested to better accommodate childcare commitments in intervention delivery. Finally, developing online, self-help resources for parents to use outside of scheduled group sessions was recommended to improve support accessibility, which would extend parental benefit beyond the lifespan of the intervention. Online resources have demonstrated acceptability and efficacy for parents of unsettled infants,^60^ further supporting this cost-effective recommendation.

The peer support intervention was acceptable both to staff and to parents. Peer support reduced feelings of self-blame and reinstated maternal confidence. For example, two of the three peer support participants noted in the evaluative interview that taking part in the intervention allowed them to attend parenting support groups with the other intervention participants, as having the presence of others with shared life experiences gave them comfort and empowered them to enjoy time with their baby despite their unsettledness (see Appendix G for supporting illustrative quotations). Regarding maternal confidence, the presence of the other peer support participants held mothers accountable to protect time for self-care activities, for example, putting on make-up in the morning because they wanted to.

Also, sharing one’s life experiences normalised their infant’s symptoms and protected their good mother identity, by allowing their infant’s symptoms to exist without judgement. Sharing personal experiences also facilitated belongingness and offered a platform for mothers to explore non-medical solutions for managing their infant’s symptoms, for example, use of blackout curtains, white noise etc., in a safe and understanding space. These findings are consistent with previous literature.^48^ Trialling a moderated online support forum specifically for parents of unsettled infants afflicted with colic, GOR(D), and/or CMPA, was proposed for future acceptability research. This recommendation was made via study insight highlighting the importance of creating a safe, non-judgemental environment for disclosure of concerns.

### Overall Study Strengths and Limitations

Co-design was a highlighted strength of the current study: harmonising the needs of parents, clinician capacity, and research pragmatism optimised project acceptability.^59^ This highlights the value of co-design in all stages of health intervention design, conduct, and write-up.^60,61^ The multidisciplinary team co-designed intervention arms which harmonised the following priorities known to determine research impact: maternal need, clinician capacity, and research pragmatism.^62^ This underscores the value of co-design at all stages of the research process and supports the integration of patient and public involvement in the development of subsequent interventions.^63^

Longitudinal, naturalistic data collection through the WhatsApp group permitted rich, in-depth understandings of caregiver experiences to be illuminated. Although the current study suffered from exceptionally poor recruitment, critical analysis of study strengths and weaknesses offer important insights for improving intervention acceptability and feasibility for research conducted with caregivers of infants with colic, GOR(D), and/or CMPA, and for engagement with healthcare services.

Secondly, the multiparous mother found study engagement more difficult alongside conflicting responsibilities, compared with primiparous mothers. This confounding factor should therefore be considered in future research endeavours. Specifically, recruitment should be stratified by parity to discern acceptability and feasibility. Finally, participant-facing documentation detracted from the informal nature of the intervention. Producing ethically sound, lay information on a research project is notoriously challenging.^64^ Including a summary leaflet in recruitment information packs would facilitate caregiver understanding of study expectations.

## Conclusion

The current study aimed to establish acceptability and feasibility of an online group peer support, health education, and music intervention to caregivers of infants with colic, GOR(D), and/or CMPA, when compared with treatment as usual. The peer support intervention was acceptable to participants and to staff. Validating one another’s experiences and normalising infantile symptoms empowered mothers and protected the good mother identity. Signposting caregivers to colic, GOR(D), and/or CMPA-specific parenting groups is recommended to enhance caregiver coping, self-efficacy, and emotional wellbeing. Evaluative focus groups identified key study strengths and limitations which can be used to inform future interventional work. Specifically, recommendations are made for an earlier point of recruitment, a study summary leaflet included in initial information packs, and for hybrid study delivery to optimise potential benefit.

## Supplemental Material

sj-docx-1-inq-10.1177_00469580251375911 – Supplemental material for Investigating the Acceptability and Feasibility of Three Online Interventions for Caregivers of Infants with Feeding Difficulties Supplemental material, sj-docx-1-inq-10.1177_00469580251375911 for Investigating the Acceptability and Feasibility of Three Online Interventions for Caregivers of Infants with Feeding Difficulties by Leanne Jackson, Ruth Drury, Giovanni Paolo Azzaro, Eduardo Coutinho, Leonardo De Pascalis, Vicky Charnock, Sian M. Davies, Clare Jones, Helen McIlroy, Sharon Remmington, Hannah Sloan, Melanie Thomas, Francine Verhoeff and Victoria Fallon in INQUIRY: The Journal of Health Care Organization, Provision, and Financing

sj-docx-10-inq-10.1177_00469580251375911 – Supplemental material for Investigating the Acceptability and Feasibility of Three Online Interventions for Caregivers of Infants with Feeding Difficulties Supplemental material, sj-docx-10-inq-10.1177_00469580251375911 for Investigating the Acceptability and Feasibility of Three Online Interventions for Caregivers of Infants with Feeding Difficulties by Leanne Jackson, Ruth Drury, Giovanni Paolo Azzaro, Eduardo Coutinho, Leonardo De Pascalis, Vicky Charnock, Sian M. Davies, Clare Jones, Helen McIlroy, Sharon Remmington, Hannah Sloan, Melanie Thomas, Francine Verhoeff and Victoria Fallon in INQUIRY: The Journal of Health Care Organization, Provision, and Financing

sj-docx-2-inq-10.1177_00469580251375911 – Supplemental material for Investigating the Acceptability and Feasibility of Three Online Interventions for Caregivers of Infants with Feeding Difficulties Supplemental material, sj-docx-2-inq-10.1177_00469580251375911 for Investigating the Acceptability and Feasibility of Three Online Interventions for Caregivers of Infants with Feeding Difficulties by Leanne Jackson, Ruth Drury, Giovanni Paolo Azzaro, Eduardo Coutinho, Leonardo De Pascalis, Vicky Charnock, Sian M. Davies, Clare Jones, Helen McIlroy, Sharon Remmington, Hannah Sloan, Melanie Thomas, Francine Verhoeff and Victoria Fallon in INQUIRY: The Journal of Health Care Organization, Provision, and Financing

sj-docx-3-inq-10.1177_00469580251375911 – Supplemental material for Investigating the Acceptability and Feasibility of Three Online Interventions for Caregivers of Infants with Feeding Difficulties Supplemental material, sj-docx-3-inq-10.1177_00469580251375911 for Investigating the Acceptability and Feasibility of Three Online Interventions for Caregivers of Infants with Feeding Difficulties by Leanne Jackson, Ruth Drury, Giovanni Paolo Azzaro, Eduardo Coutinho, Leonardo De Pascalis, Vicky Charnock, Sian M. Davies, Clare Jones, Helen McIlroy, Sharon Remmington, Hannah Sloan, Melanie Thomas, Francine Verhoeff and Victoria Fallon in INQUIRY: The Journal of Health Care Organization, Provision, and Financing

sj-docx-4-inq-10.1177_00469580251375911 – Supplemental material for Investigating the Acceptability and Feasibility of Three Online Interventions for Caregivers of Infants with Feeding Difficulties Supplemental material, sj-docx-4-inq-10.1177_00469580251375911 for Investigating the Acceptability and Feasibility of Three Online Interventions for Caregivers of Infants with Feeding Difficulties by Leanne Jackson, Ruth Drury, Giovanni Paolo Azzaro, Eduardo Coutinho, Leonardo De Pascalis, Vicky Charnock, Sian M. Davies, Clare Jones, Helen McIlroy, Sharon Remmington, Hannah Sloan, Melanie Thomas, Francine Verhoeff and Victoria Fallon in INQUIRY: The Journal of Health Care Organization, Provision, and Financing

sj-docx-5-inq-10.1177_00469580251375911 – Supplemental material for Investigating the Acceptability and Feasibility of Three Online Interventions for Caregivers of Infants with Feeding Difficulties Supplemental material, sj-docx-5-inq-10.1177_00469580251375911 for Investigating the Acceptability and Feasibility of Three Online Interventions for Caregivers of Infants with Feeding Difficulties by Leanne Jackson, Ruth Drury, Giovanni Paolo Azzaro, Eduardo Coutinho, Leonardo De Pascalis, Vicky Charnock, Sian M. Davies, Clare Jones, Helen McIlroy, Sharon Remmington, Hannah Sloan, Melanie Thomas, Francine Verhoeff and Victoria Fallon in INQUIRY: The Journal of Health Care Organization, Provision, and Financing

sj-docx-6-inq-10.1177_00469580251375911 – Supplemental material for Investigating the Acceptability and Feasibility of Three Online Interventions for Caregivers of Infants with Feeding Difficulties Supplemental material, sj-docx-6-inq-10.1177_00469580251375911 for Investigating the Acceptability and Feasibility of Three Online Interventions for Caregivers of Infants with Feeding Difficulties by Leanne Jackson, Ruth Drury, Giovanni Paolo Azzaro, Eduardo Coutinho, Leonardo De Pascalis, Vicky Charnock, Sian M. Davies, Clare Jones, Helen McIlroy, Sharon Remmington, Hannah Sloan, Melanie Thomas, Francine Verhoeff and Victoria Fallon in INQUIRY: The Journal of Health Care Organization, Provision, and Financing

sj-docx-7-inq-10.1177_00469580251375911 – Supplemental material for Investigating the Acceptability and Feasibility of Three Online Interventions for Caregivers of Infants with Feeding Difficulties Supplemental material, sj-docx-7-inq-10.1177_00469580251375911 for Investigating the Acceptability and Feasibility of Three Online Interventions for Caregivers of Infants with Feeding Difficulties by Leanne Jackson, Ruth Drury, Giovanni Paolo Azzaro, Eduardo Coutinho, Leonardo De Pascalis, Vicky Charnock, Sian M. Davies, Clare Jones, Helen McIlroy, Sharon Remmington, Hannah Sloan, Melanie Thomas, Francine Verhoeff and Victoria Fallon in INQUIRY: The Journal of Health Care Organization, Provision, and Financing

sj-docx-8-inq-10.1177_00469580251375911 – Supplemental material for Investigating the Acceptability and Feasibility of Three Online Interventions for Caregivers of Infants with Feeding Difficulties Supplemental material, sj-docx-8-inq-10.1177_00469580251375911 for Investigating the Acceptability and Feasibility of Three Online Interventions for Caregivers of Infants with Feeding Difficulties by Leanne Jackson, Ruth Drury, Giovanni Paolo Azzaro, Eduardo Coutinho, Leonardo De Pascalis, Vicky Charnock, Sian M. Davies, Clare Jones, Helen McIlroy, Sharon Remmington, Hannah Sloan, Melanie Thomas, Francine Verhoeff and Victoria Fallon in INQUIRY: The Journal of Health Care Organization, Provision, and Financing

sj-docx-9-inq-10.1177_00469580251375911 – Supplemental material for Investigating the Acceptability and Feasibility of Three Online Interventions for Caregivers of Infants with Feeding Difficulties Supplemental material, sj-docx-9-inq-10.1177_00469580251375911 for Investigating the Acceptability and Feasibility of Three Online Interventions for Caregivers of Infants with Feeding Difficulties by Leanne Jackson, Ruth Drury, Giovanni Paolo Azzaro, Eduardo Coutinho, Leonardo De Pascalis, Vicky Charnock, Sian M. Davies, Clare Jones, Helen McIlroy, Sharon Remmington, Hannah Sloan, Melanie Thomas, Francine Verhoeff and Victoria Fallon in INQUIRY: The Journal of Health Care Organization, Provision, and Financing
