# Supplementary material for: Investigating the Acceptability and Feasibility of Three Online Interventions for Caregivers of Infants with Feeding Difficulties
Source: Inquiry. 2025 Oct 18;62:00469580251375911. doi: 10.1177/00469580251375911 (PMC12547111; doi:10.1177/00469580251375911)
Supplement: sj-docx-1-inq-10.1177_00469580251375911 – Supplemental material for Investigating the Acceptability and Feasibility of Three Online Interventions for Caregivers of Infants with Feeding Difficulties [file sj-docx-1-inq-10.1177_00469580251375911.docx]

Supplementary materials: CONSORT statement

| **Section/topic** | **Item number** | **Checklist item** | **Where detailed in manuscript** |
| --- | --- | --- | --- |
| Title and abstract | 1a | Identification as a randomised trial in the title | Study was not a randomised trial, but title identifies it as a feasibility study, “Investigating the acceptability and feasibility of three online interventions, for caregivers of infants with feeding difficulties” |
|  | 1b | Structured summary of trial design, methods, results, and conclusions | See abstract, “**Methods:** Eligible caregivers were recruited during routine appointments with the infant feeding team at Alder Hey Children’s NHS Foundation Trust. Consenting caregivers were assigned to one of the four intervention arms. For peer support only, a WhatsApp group accompanied group sessions. Intervention weeks 1-3 involved a one-hour online group session, where skills were developed with an aim to improve management of infantile symptoms, and to nurture self-care practices. In weeks 4-6, participants were encouraged to use skills obtained from weeks 1-3, independently. In week 7, evaluative focus groups were conducted. WhatsApp group data underwent conversational analysis and evaluative focus group data underwent thematic analysis. **Results:** Feasibility was not achieved due to recruitment difficulties. However, the peer support intervention was deemed acceptable by mothers and staff. Peer support participants valued the flexibility access to support via WhatsApp with other mothers with shared life experience. **Conclusion:** Evaluative focus groups identified study strengths and limitations which will provide insight to digital health researchers seeking to develop interventional research for caregivers of infants afflicted with colic, GOR(D), and/or CMPA.” |
| Introduction  Background and objectives | 2a | Scientific background and explanation of the rationale | See Section 1, ‘Introduction’. |
|  | 2b | Specific objectives or hypotheses | For operationalised objectives and hypotheses, see section 1.4 of the introduction, ‘Current study’ |
| Methods  Trial design | 3a | Description of trial design (such as parallel, factorial) including allocation ratio | Details on intervention design can be found in section 2.4 of the methods. Appendices A-D also provide more detailed information on set up of the respective intervention arms and highlights where standardising efforts have been made. See section 2.4 for information on pragmatic sampling and group allocation. See section 2.2. Study design, “The current pilot study aimed to investigate the feasibility and acceptability of an online group peer support, health education, and music intervention for caregivers of infants affected by colic, reflux, and GOR(D).  Study is identified as a pilot study to investigate the acceptability and feasibility of three group online interventions throughout the introduction and methods sections of the manuscript. |
|  | 3b | Important changes to methods after trial commencement (such as eligibility criteria), with reasons | Not applicable. No changes were made to the methods after trial commencement. |
| Participants | 4a | Eligibility criteria for participants | This information can be found in section 2.3 ‘Participants’ sub-section of the methods, “Mothers who had been referred to Alder Hey Children’s NHS Foundation Trust (Hereafter referred to as ‘Alder Hey’) with their unsettled infant, due to infant feeding difficulties relating to Colic, GOR(D), and/or CMPA, were identified by clinicians employed at Alder Hey. All caregivers who met the following eligibility criteria were  approached by their clinician during routine appointments: over the age of 18, and no previous or current clinical diagnosis of a  serious mental health condition i.e., bipolar disorder, schizophrenia, and/or psychosis (confirmed via self-report on initial contact [LJ], described below). Infants needed to be  younger than six months old at time of referral, born full-term (i.e., >34 weeks' gestation), without known comorbidities or  evidence of faltering growth^34^ for their caregiver to be eligible to participate. |
|  | 4b | Settings and locations where the data were collected | Not applicable – participants were recruited from one site, only. Information on eligibility criteria and recruitment process can be found in section 2.3 ‘Participants’ of the methods in the current manuscript. All participants were recruited by their healthcare practitioners,  Employed by Alder Hey. Full details on the recruitment process and data collection can be found in sections 2.3, 2.4, and 2.6 of  The methods in the current manuscript. |
| Interventions | 5 | The interventions for each group with sufficient details to allow replication, including how and when they were actually administered | Full information on set up of the intervention arms, including where design has been standardised can be found in section 2.4 of the manuscript’s methods. See appendices for more detailed break down of the respective intervention arms. PowerPoint slides from each intervention arm can also be made available on reasonable request. |
| Outcomes | 6a | Completely defined pre-specified primary and secondary outcome measures, including how and when they were assessed | Psychometric measures administered at baseline and in week 6 to assess change over time in wellbeing measures, which are detailed in section 2.5’ pre-and post-assessment questionnaires’ section of the methods. Evaluative focus groups were also  conducted in week 7 to investigate lived experiences of taking part in the pilot study, to inform design in scale up of the  interventions. |
|  | 6b | Any changes to trial outcomes after the trial commenced, with reasons | Not applicable. No changes were made to the trial outcomes after trial commencement. |
| Sample size | 7a | How sample size was determined? | A pragmatic sample of 40 participants was sought, as is common in feasibility research. This information can be found in section 2.4 ‘Group allocation and intervention details’  sub-section of the methods. |
|  | 7b | When applicable, explanation of any interim analyses and stopping guidelines | Interim questionnaires were administered on the Friday of each intervention week to assess usefulness of each intervention week.  However, due to poor completion rates, these measures were not analysed in final write up of the study. This information can be found in section, ‘2.*7.1 pre-, post- and interim assessment measures’* of the methods. |
| Randomization  Sequence generation | 8a | The method used to generate the random allocation sequence | Not applicable. See section 2.4 ‘Group allocation and intervention details’ sub-section of the methods for details on  sampling method. |
|  | 8b | Type of randomization; details of any restriction (such as blocking and block size) | Not applicable. See section 2.4 ‘Group allocation and intervention details’ sub-section of the methods for details on  sampling method. |
| Allocation concealment mechanism | 9 | The mechanism used to implement the random allocation sequence (such as sequentially numbered containers), describing any steps taken to conceal the sequence until interventions were assigned | Not applicable. See section 2.4, “Treatment as usual ran first due to lack of prior preparation required to set up the intervention arm. Clinician capacity meant that six weeks’ notice was required to block out sufficient time to run the health education intervention arm alongside their usual caseloads. Therefore, the health education intervention arm ran third, and the peer support intervention arm ran second due to lack of pre-preparations needed to run this intervention arm. Music intervention ran last due to capacity of the employed harpist, who facilitated scheduled group intervention sessions.” |
| Implementation | 10 | Who generated the random allocation sequence, who enrolled participants, and who assigned participants to interventions | Not applicable. See section 2.4 ‘Group allocation and intervention details’ sub-section of the methods for details on sampling method. |
| Blinding | 11a | If done, who was blinded after assignment to interventions (e.g., participants, care providers, those assessing outcomes) and how | Details of intervention arm design can be found in the following section of the methods, “*2.4 Group allocation and intervention details”* including information on how the intervention arms were standardised where it has been possible to do so. More detailed information on the respective intervention arms can also be found in Appendices A-D. PowerPoint slides for the respective intervention arms can also be made available on reasonable request.  See section 2.4 for more information on participant allocation to intervention arms, “Treatment as usual ran first due to lack of prior preparation required to set up the intervention arm. Clinician capacity meant that six weeks’ notice was required to block out sufficient time to run the health education  intervention arm alongside their usual caseloads. Therefore, the health education intervention arm ran third, and the peer support intervention arm ran second due to lack of pre-preparations needed to run this intervention arm. Music intervention ran last  due to capacity of the employed harpist, who facilitated scheduled group intervention sessions.” |
|  | 11b | If relevant, description of the similarity of interventions | Details of intervention arm design can be found in the following section of the methods, “*2.4 Group allocation and intervention details”* including information on how the intervention arms were standardised where it has been possible to do so. More detailed information on the respective intervention arms can also be found in Appendices A-D. PowerPoint slides for the respective intervention arms can also be made available on reasonable request. |
| Statistical methods | 12a | Statistical methods used to compare groups for primary and secondary outcomes | Inferential statistics were not performed on  administered psychometric measures (pre-post comparison or interim assessments) due to exceptionally poor completion rates. See section 3 ‘Integrated results and discussion section’ for discussion of findings from the conversation analysis and reflexive thematic analysis, from collected qualitative data. |
|  | 12b | Methods for additional analyses, such as subgroup analyses and adjusted analyses | Not applicable – see above. |
| Results  Participant flow (a diagram is strongly recommended) | 13a | For each group, the numbers of participants who were randomly assigned received intended treatment and were analyzed for the primary outcome | Not applicable – see above. See section 2.4 for information on the number of participants included in the final sample for each intervention arm. |
|  | 13b | For each group, losses and exclusions after randomization, together with reasons | Information on participant attrition and final sample size per intervention group can be found in section 2.4 ‘Group allocation  and intervention details’ in the methods of the manuscript. |
| Recruitment | 14a | Dates defining the periods of recruitment and follow-up | Information on periods of recruitment can be found in section 2.4 ‘Group allocation and intervention details’ in the methods of the manuscript. |
|  | 14b | Why the trial ended or was stopped | Information on participant recruitment period, attrition, final sample size per intervention group, and why the recruitment  period was not extended can be found in section 2.4 ‘Group allocation and intervention details’ in the methods of the  manuscript. |
| Baseline data | 15 | A table showing the baseline demographic and clinical characteristics for each group | See Table 1. |
| Numbers analyzed | 16 | For each group, number of participants (denominator) included in each analysis and whether the analysis was by original assigned groups | Not applicable – inferential statistics were not performed on administered psychometric measures (pre-post comparison or interim assessments) due to exceptionally poor completion rates. Information on this can be found in section 2.7 ‘Analysis’ sub-section of the methods. A conversation analysis (see section 2.7.2 of the methods) was conducted on the WhatsApp group data collected for the peer support intervention arm and a reflexive thematic analysis was conducted on evaluative focus group data (see section 2.7.3 for details in the methods of the  manuscript for more information). |
| Outcomes and estimation | 17a | For each primary and secondary outcome, results for each group, and the estimated effect size and its precision (such as 95% confidence interval) | Inferential statistics were not performed on  administered psychometric measures (pre post comparison or interim assessments) due to exceptionally poor completion rates. See section 3 ‘Integrated results and discussion section’ for discussion of findings from the conversation analysis and reflexive thematic analysis, from collected qualitative data. |
|  | 17b | For binary outcomes, presentation of both absolute and relative effect sizes is recommended | Inferential statistics were not performed on  administered psychometric measures (pre post comparison or interim assessments) due to exceptionally poor completion rates. See section 3 ‘Integrated results and discussion section’ for discussion of findings from the conversation analysis and reflexive thematic analysis, from collected qualitative data. |
| Ancillary analyses | 18 | Results of any other analyses performed, including subgroup analyses and adjusted analyses, distinguishing pre-specified from exploratory | See section 3 ‘Integrated results and discussion section’ for discussion of findings from the conversation analysis and reflexive thematic analysis, from collected qualitative data. Also see Figure 1, Table 2, and appendices G and H for illustrative quotations and supporting evidence for qualitative findings and discussion. |
| Harms | 19 | All-important harms or unintended effects in each group (for specific guidance see CONSORT for harms) | See section 2.1 ‘Ethical statement’ for consideration of participant harm in study set up. |
| Discussion Limitations | 20 | Trial limitations, addressing sources of potential bias, imprecision, and, if relevant, the multiplicity of analyses | See section 3 ‘Integrated results and discussion section’ for extensive discussion of study limitations and suggestions for improvements. |
| Generalizability | 21 | Generalizability (external validity, applicability) of the trial findings | See section 3 ‘Integrated results and discussion section’ for extensive discussion of these points. |
| Interpretation | 22 | Interpretation consistent with results, balancing benefits and harms, and considering other relevant evidence | See section 3 ‘Integrated results and discussion section’ for interpretation of findings in relation to relevant literature, consideration of benefits and harms and providing insights for future research. |
| Other information  Registration | 23 | Registration number and name of trial registry | See section 2.2 Study design and title page, “The present study was pre-registered through the International Standard Randomised Controlled Trial Number (ISRCTN) registry on 06 Aug 2021 (ID: ISRCTN15349263). |
| Protocol | 24 | Where the full trial protocol can be accessed, if available | See section 2.2 Study design and title page, “The present study was pre-registered through the International Standard Randomised Controlled Trial Number (ISRCTN) registry on 06 Aug 2021 (ID: ISRCTN15349263).” The full study protocol can be found here. |
| Funding | 25 | Sources of funding and other support (such as the supply of drugs), the role of funders | Funding statement can be found on the title page of the manuscript and at the end of the manuscript, “This study was funded by the Hugh Greenwood Legacy for Children’s Health Research (Ref: C14). Please note that the funder was not involved in the set-up, conduct, or write up of the current  project as submitted for publication.” |
