# Supplementary material for: Investigating the Acceptability and Feasibility of Three Online Interventions for Caregivers of Infants with Feeding Difficulties
Source: Inquiry. 2025 Oct 18;62:00469580251375911. doi: 10.1177/00469580251375911 (PMC12547111; doi:10.1177/00469580251375911)
Supplement: sj-docx-10-inq-10.1177_00469580251375911 – Supplemental material for Investigating the Acceptability and Feasibility of Three Online Interventions for Caregivers of Infants with Feeding Difficulties [file sj-docx-10-inq-10.1177_00469580251375911.docx]

**Author reflexivity statement**

XX is a Master’s student in clinical Psychology at University La Sapienza, Rome. English was XX’s second language, which he found challenging when translating audio files and interpreting colloquialisms during analysis. All transcribed audio files were checked for accuracy by an independent researcher who was otherwise unaffiliated with the current study, and blind to its aims [XX]. Any inconsistencies and/or inaccuracies were mended at this point. Additionally, a perinatal researcher with extensive experience in qualitative research methods co-analysed the WhatsApp data [XX], to ensure that final thematic structures were appropriately embedded in participant accounts^48-49^. In one respect, XX considered that being male may have limited their ability to empathise with maternal accounts as deeply as a female analyst. However, having a sibling who had been afflicted with GORD enabled a deeper level of understanding to be held towards participants affected by the conditions under investigation.

Thematic analysis was conducted by XX, a Psychology Conversion MSc student at the [University]. XX is a nulliparous cis-female who does not have any clinical training or personal experience related to colic, GOR(D), and/or CMPA. This lack of direct experience was viewed as an advantage as it allowed XX to analyse with greater objectivity and minimal personal biases. Conversation and thematic analyses were co-analysed by XX, who is an early career researcher in perinatal mental health with extensive experience in qualitative research methods. XX does not have clinical training, which allows for an objective stance to be taken during conversations about infant symptomatology and treatment. Final thematic structures were revised and agreed upon by all members of the research team, including perinatal and music academics [XX, XX, XX, XX], clinical staff with neonatal, infant feeding, and maternity expertise [XX, XX, XX, XX, XX, XX], and a parent with relevant lived experience [XX].
