# Supplementary material for: Investigating the Acceptability and Feasibility of Three Online Interventions for Caregivers of Infants with Feeding Difficulties
Source: Inquiry. 2025 Oct 18;62:00469580251375911. doi: 10.1177/00469580251375911 (PMC12547111; doi:10.1177/00469580251375911)
Supplement: sj-docx-2-inq-10.1177_00469580251375911 – Supplemental material for Investigating the Acceptability and Feasibility of Three Online Interventions for Caregivers of Infants with Feeding Difficulties [file sj-docx-2-inq-10.1177_00469580251375911.docx]

**Appendix A:**

**Protocol (Treatment As Usual; TAU)**

**IRAS ID: 296579**

**Version 1.1, 15^th^ April 2021**

**Intervention**

**Duration:** 6 weeks

**Procedure**:

- Referral into infant feeding clinic after review by Health visitor, GP, emerging department Clinician or General paediatrician.

**Treatment as usual-specific measures (before, during and after the intervention)**

***Pre- and post-assessment questionnaires***

Firstly, participants will answer a few demographic questions about their home composition, age, infant date of birth, the first three characters of their postcode (to determine socioeconomic status), and their infant feeding method. Infant feeding method will be assessed using a validated 11-point Likert Scale with percentage response options varying from 100% formula fed to 100% breastfed over the past 48-hour period (Davie, 2018).

*Perceived Maternal Parenting Self-Efficacy (PMPSE) tool (Barnes & Adamson-Macedo, 2007).*

20-item self-report questionnaire to assess perceived parenting self-efficacy with four sub-scales reflecting different parenting domains: care taking procedures, evoking behaviour(s), reading behaviour(s) or signalling, and situational beliefs. Response options include, ‘strongly disagree’, ‘disagree’, ‘agree’ and ‘strongly agree’. Higher scores on this questionnaire reflect higher perceived parenting self-efficacy.

*Edinburgh Postnatal Depression Scale (EPDS; Cox et al., 1987)*

10-item self-report questionnaire administered to screen for depressive symptoms in the postnatal period. It is the most widely used screening scale for postnatal depression. Higher scores indicate higher levels of depression. A clinical cut-off score of ≥13 identifies scores consistent with major depressive disorder, although the self-report measure does not replace a clinical diagnosis.

*Postpartum Specific Anxiety Scale (PSAS; Fallon et al, 2021)*

*16-item self-report questionnaire to assess perceived parenting anxiety in the postpartum period. Questionnaire items cover four domains of parenting: psychosocial adjustment to motherhood anxieties, practical infant care anxieties, maternal competence and attachment anxieties, and infant safety and welfare anxieties. Higher scores indicate higher levels of anxiety. Measured using 4 point Likert scale response options from '0 Not at all' to '3 Almost Always'.*

*Short Assessment of Patient Satisfaction (SAPS;* Hawthorne et al, 2014)

7-item self-report questionnaire to assess perceived satisfaction with healthcare professional support. Response options include, ‘very satisfied’, ‘satisfied’, ‘Neither satisfied nor dissatisfied’, ‘Dissatisfied’, and, ‘Very dissatisfied’. Higher scores on this scale correspond with greater perceived satisfaction with healthcare professional support.

Researcher-developed items, tailored for TAU:

1. Did your midwife discuss different forms of feeding for your baby with you? Yes/No
2. Did you find this information useful? Yes/No
3. Other than the Hospital infant feeding team, who have you sought infant feeding advice from for your unsettled/distressed baby? Midwife/Health visitor/GP/Walk-in Centre/Hospital Emergency Department/Paediatrician/Other

**AFTER** the intervention, it would be also relevant to measure the perceived effectiveness of treatment as usual

Same measures that were administered before the intervention (1) are to be administered again to assess change over time and effectiveness of treatment as usual.

**First appointment**

**Duration:** 30 mins

**Timing:** Start of Week 1

**Aims:**

- First appointment generally face-to-face. Assessment made, advice given, and family directed to Hospital web site for the relevant patient information leaflets (Hospital, N/Aa; Hospital, N/Ab).
- Follow-up appointment, if required, often video or telephone consultation.

Follow-up appointment(s) then organised as required.

Discharge back to universal services and primary care when input from specialist services no longer required.

**Focus group**

**Duration:** 1 hour

**Timing:** After the intervention (start of Week 7)

- Discuss relevant issues related to treatment as usual for feeding back into clinic service evaluation e.g. accessibility of service, effectiveness of communication with healthcare practitioner(s), evaluation of virtual care compared with face-to-face care.

**References**

[Reference removed due to identifiable content for Hospital site]

[Reference removed due to identifiable content for Hospital site]

Barnes, C. R. & Adamson-Macedo, E. N. (2007). Perceived Maternal Parenting Self-Efficacy (PMP S-E) tool: Development and validation with mothers of hospitalized preterm neonates. JAN Research Methodology, 60(5), 550-561

Cox, J.L., Holden, J.M., Sagovsky, R., 1987. Detection of postnatal depression: development of the 10-item Edinburgh postnatal depression scale. Br. J. Psychiatr. 150 (6), 782–786. https://doi.org/10.1192/bjp.150.6.782

Davie, P (2018). Measuring milk: A call for change in quantifying breastfeeding behaviour. *Midwifery, 63,* 6-7

Royal College of Pediatrics and Child Health (RCPCH; N/A). <https://www.rcpch.ac.uk/sites/default/files/PaedCCF_Example_Form_OP.pdf>

Hawthorne, G., Sansoni, J., Hayes, L., Marosszeky, N., & Sansoni, E. (2014). Measuring patient satisfaction with health care treatment using the Short Assessment of Patient Satisfaction measure delivered superior and robust satisfaction estimates. Journal of clinical epidemiology, 67(5), 527-537.

Taylor, A., Atkins, R., Kumar, R., Adams, D., Glover, V., 2005. A new Mother-to-Infant Bonding Scale: links with early maternal mood. Arch. Wom. Ment. Health 8 (1), 45–51. <https://doi.org/10.1007/s00737-005-0074-z>
