# Supplementary material for: Investigating the Acceptability and Feasibility of Three Online Interventions for Caregivers of Infants with Feeding Difficulties
Source: Inquiry. 2025 Oct 18;62:00469580251375911. doi: 10.1177/00469580251375911 (PMC12547111; doi:10.1177/00469580251375911)
Supplement: sj-docx-3-inq-10.1177_00469580251375911 – Supplemental material for Investigating the Acceptability and Feasibility of Three Online Interventions for Caregivers of Infants with Feeding Difficulties [file sj-docx-3-inq-10.1177_00469580251375911.docx]

**Appendix B:**

**Protocol (peer support)**

**IRAS ID: 296579**

**Version 1.3, 15^th^ April 2021**

**Intervention**

**Duration:** 6 weeks

**Procedure**:

- Participate in 3 weekly groups sessions (see below for content) in Weeks 1-3
- Ask carers to use WhatsApp group for emotional support and social interaction only, and to contact their health professional for medical guidance. Remind participants that their posts in the chat will be anonymised and analysed, unless the participant indicates through an emoji that they would like their post to be excluded from analysis.

**Peer support-specific measures (before, during and after the intervention)**

***Pre-intervention only***

1. Firstly, participants will answer a few demographic questions about their home composition, age, infant date of birth, the first three characters of their postcode (to determine socioeconomic status), and their infant feeding method. Given that the engagement with the WhatsApp support group may be related to past social media experiences and value attributed to peer support it would be important, therefore, for carers to be administered a social media usage history questionnaire **BEFORE** the intervention starts, only.

- Do you use online parenting support groups to support you when your baby is unsettled? Yes/No.
- How often did you use online parenting support groups in the last 48 hours? Not at all/1-2 times a day/3-5 times a day/>5 times a day
- Have you found using parenting support groups helpful in soothing your baby’s symptoms when they are unsettled? Most of the time/Often/Sometimes/Never
- Did you experience positive feelings whilst using online parenting support groups to help support you with your unsettled baby? Most of the time/Often/Sometimes/Never
- Please complete this statement: My baby is conent… Most of the time/Often/Sometimes/Never

The next question will be assessed using 5 point Likert-scale response options:

Please complete this statement: When my baby is unsettled I feel…

1 Useless 2 3 4 5 Able to cope

1 Anxious 2 3 4 5 Calm

1 Guilty 2 3 4 5 Not Guilty

1 Lonely 2 3 4 5 Supported

1 Frustrated 2 3 4 5 Patient

***Pre- and post-assessment questionnaires***

To examine the effectiveness of the intervention condition, the following questionnaires will be administered before and after the 6-week intervention involvement in this study:

Infant feeding method will be assessed using a validated 11-point Likert Scale with percentage response options varying from 100% formula fed to 100% breastfed over the past 48-hour period (Davie, 2018).

*Perceived Maternal Parenting Self-Efficacy (PMPSE) tool (Barnes & Adamson-Macedo, 2007).*

20-item self-report questionnaire to assess perceived parenting self-efficacy with four sub-scales reflecting different parenting domains: care taking procedures, evoking behaviour(s), reading behaviour(s) or signalling, and situational beliefs. Response options include, ‘strongly disagree’, ‘disagree’, ‘agree’ and ‘strongly agree’. Higher scores on this questionnaire reflect higher perceived parenting self-efficacy.

*Edinburgh Postnatal Depression Scale (EPDS; Cox et al., 1987)*

10-item self-report questionnaire administered to screen for depressive symptoms in the postnatal period. It is the most widely used screening scale for postnatal depression. Higher scores indicate higher levels of depression. A clinical cut-off score of ≥13 identifies scores consistent with major depressive disorder, although the self-report measure does not replace a clinical diagnosis.

*Postpartum Specific Anxiety Scale (PSAS; Fallon et al, 2021)*

16-item self-report questionnaire to assess perceived parenting anxiety in the postpartum period. Questionnaire items cover four domains of parenting: psychosocial adjustment to motherhood anxieties, practical infant care anxieties, maternal competence and attachment anxieties, and infant safety and welfare anxieties. Higher scores indicate higher levels of anxiety. Measured using 4 point Likert scale response options from '0 Not at all' to '3 Almost Always'.

*Short Assessment of Patient Satisfaction (SAPS; Hawthorne et al, 2014)*

7-item self-report questionnaire to assess perceived satisfaction with healthcare professional support. Response options include, ‘very satisfied’, ‘satisfied’, ‘Neither satisfied nor dissatisfied’, ‘Dissatisfied’, and, ‘Very dissatisfied’. Higher scores on this scale correspond with greater perceived satisfaction with healthcare professional support.

*Please rate how much to you agree with each of the following statements using a scale ranging from 0 (completely disagree) to 10 (completely agree)*

- I use online parenting support groups about my baby’s distress
- I think it is very important to use online parenting support groups about my baby’s distress
- I experience positive feelings when using online parenting support groups
- Accessing online parenting support groups helps me to manage by baby better
- Accessing online parenting support groups helps me to manage my baby when he/she is distressed

***During all intervention weeks (1-6):***

1. **DURING** the intervention, to quantify engagement with the virtual support group and the state of the infant and the carer it would be very important to monitor the following variables on a weekly basis:
   - *How often did you use the online parenting support group over the past week?*
     - *Every day, most days, about half of the week, rarely, never*

Perception of infant’s symptoms that week (e.g., amount of crying, general distress, hours of sleep, etc.

- Did you feel that using the online parenting support group has given you support to soothe your baby when he/she was distressed? Always, Usually, About half the times, Rarely, Never
- Did you feel that using the WhatsApp support group helped you to cope and feel better, generally? Always, Usually, About half the times, Rarely, Never
- Did you feel that using the WhatsApp group has helped you to cope and feel better, when your baby was distressed? Always, Usually, About half the times, Rarely, Never

***Post-intervention involvement (week 6):***

1. **AFTER** the intervention, it would be also relevant to measure the perceived importance of peer support. Same measures that were administered before the intervention (1) are to be administered again to assess change over time and effectiveness of the intervention.

**First group session**

**Duration:** 1 hour

**Timing:** Start of Week 1

**Aims:**

- 35-minute introduction

To inform carer about what peer support is

To inform carer about the benefits of peer support (use success stories from other projects)

To provide an overview of the WhatsApp group

The carer will be briefed on ground rules for using the peer support WhatsApp group i.e., what can and cannot be posted on the group, how breaches of these rules will be handled, group confidentiality and online safety (e.g., use of pseudonyms if desired), and how one can choose to exclude posts from analysis.

Caregivers will be encouraged to engage in conversation in response to pro-active posts by the research team and will be encouraged to use the group as and when needed between these check-ins. Particular focus will be placed on the importance of shared experiences and emotional support/connection in improving caregiver wellbeing and coping.

5 minutes for any questions about introductory information.

Moderating member of the research team will set up a WhatsApp parenting group, using mobile numbers provided in the consent forms for this intervention arm. As a ‘homework task, caregivers will be asked to introduce themselves to the WhatsApp group:

- - This can include encouraging mothers to talk about what they would like to gain from the group (Trickey et al, 2017) and introducing their infant to the group before the next session e.g., one cute fact. It will be emphasised that engagement is voluntary, throughout all intervention sessions.
  - What techniques they currently use to soothe their baby when he/she is distressed.

**Second group session**

**Duration:** 1 hour

**Timing:** Start of Week 2

**Aims:**

- 20 minutes [PPIE blinded for review] representative to talk about personal success stories relevant to infant colic and reflux (Regan & Brown, 2019).
- 15 minutes time for informal discussion between attendees e.g., how they’re getting on with baby’s symptoms, emotionally, how engaging with support group (field notes to be taken by research assistant).
- 5 minute ‘WhatsApp engager’ (ask mothers to share a new hobby/activity they have picked up during lockdown in the parenting group to boost engagement in the WhatsApp group).

**Third group session**

**Duration:** 1 hour

**Timing:** Start of Week 3

**Aims:**

- To follow-up on the first two sessions, identify potential problems/difficulties, increase confidence, etc. Is there anything that we could have done to improve your experience?
- Set groups off with ‘engager’ activity to share experiences.

**Focus group**

**Duration:** 1 hour

**Timing:** After the intervention (start of Week 7)

- Discuss relevant issues related to interventions (e.g., experience, perceived benefits, barriers, etc.)

**WhatsApp group (throughout intervention)**

**Aims:** To encourage a sense of group belongingness and increase feelings of emotional support between group sessions. To improve caregiver self-confidence and general wellbeing through group cohesion and shared experiences. Prompts are to be posted by the moderating member of the research team on a weekly basis, adopting a pro-active approach to peer support exchanges (Martinez-Brockman et al, 2019).

The group will be moderated by one member of the research team to ensure that group rules are being adhered to (Regan & Brown, 2019). This moderating member of the research team will be briefed on the disclosure of health ‘red flags’ which would need professional referral prior to setting up the group chat (Merewood & Philipp, 2003). After reiterating the ground rules for using the WhatsApp group, the following prompt schedule will be implemented by the moderating member of the research team:

**Week 1** “Welcome to the group. Let’s get to know each other! Post your favourite mum and baby meme and share a little bit about your experience with your baby’s colic/reflux.’

**Week 2 “**What were your thoughts on the speaker in this week’s group session? Was there anything that came up in the session, that you haven’t tried before, or that you think might help? Do you identify with what was talked about?”

**Week 3** “What are your favourite self-care activities? Share something that you plan on doing to take some time out for yourself this next week!”

**Week 4** “Using only a gif, share how your week has been!”

**Week 5** “It’s Friday! Does anyone have any nice plans for the weekend?”

**Week 6** “Tell me something positive that you are going to take away from being part of this support group!”

The WhatsApp group will also be used to send a reminder about completing weekly interim surveys and post-intervention surveys at the end of week 6.

**References**

Barnes, C. R. & Adamson-Macedo, E. N. (2007). Perceived Maternal Parenting Self-Efficacy (PMP S-E) tool: Development and validation with mothers of hospitalized preterm neonates. JAN Research Methodology, 60(5), 550-561

Cevasco, A. M. (2008). The effects of mothers' singing on full-term and preterm infants and maternal emotional responses. Journal of music therapy, 45(3), 273-306

Cox, J.L., Holden, J.M., Sagovsky, R., 1987. Detection of postnatal depression: development of the 10-item Edinburgh postnatal depression scale. Br. J. Psychiatr. 150 (6), 782–786. <https://doi.org/10.1192/bjp.150.6.782>

Davie, P (2018). Measuring milk: A call for change in quantifying breastfeeding behaviour. *Midwifery, 63,* 6-7

Gibaud-Wallston, J., Wandersmann, L.P., (1978). Development and utility of the Parenting Sense of Competence Scale. Paper presented at the annual meeting of the American Psychological Association, Toronto, Canada. John F. Kennedy Center for Research on Education and Human Development

Martinez-Brockman, J. L., Harari, N., Goeschel, L., Bozzi, V., & Pérez-Escamilla, R. (2019). A qualitative analysis of text message conversations in a breastfeeding peer counselling intervention. Maternal & Child Nutrition, 1-13. https://doi.org/10.1111/mcn.12904

Regan, S. & Brown, A. (2019). Experiences of online breastfeeding support: Support and reassurance versus judgement and misinformation. Maternal and Child Nutrition, 1-13. https://doi.org/10.1111/mcn.12874

Merewood, A. & Philipp, B. L. (2003). Peer Counselors for Breastfeeding Mothers in the Hospital Setting: Trials, Training, Tributes, and Tribulations. Journal of Human Lactation, 19(1), 72-76

Silverio, S. A., Davies, S. M., Christiansen, P., Aparicio-García, M. E., Bramante, A., Chen, P., Costas-Ramón, N., De Weerth, C., Vedova, A. M. D., Gil, L. I., Lustermans, H., Wendland, J., Xu, J., Halford, J. C. G., Harrold, J. A., & Fallon, V. (2021). A validation of the Postpartum Specific Anxiety Scale 12-item research short-form for use during global crises with five translations. BMC Pregnancy and Childbirth, 21, 1-12. https://doi.org/10.1186/s12884-021-03597-9

Taylor, A., Atkins, R., Kumar, R., Adams, D., Glover, V., 2005. A new Mother-to-Infant Bonding Scale: links with early maternal mood. Arch. Wom. Ment. Health 8 (1), 45–51. <https://doi.org/10.1007/s00737-005-0074-z>

Wynter, K., Hammarberg, K., Sartore, G. M., Cann, W., & Fisher, J. (2015). Brief online surveys to monitor and evaluate facilitated peer support groups for caregivers of children with special needs. *Evaluation and Program Planning, 49,* 70-75. <http://dx.doi.org/10.1016/j.evalprogplan.2014.12.019>
