# Supplementary material for: Investigating the Acceptability and Feasibility of Three Online Interventions for Caregivers of Infants with Feeding Difficulties
Source: Inquiry. 2025 Oct 18;62:00469580251375911. doi: 10.1177/00469580251375911 (PMC12547111; doi:10.1177/00469580251375911)
Supplement: sj-docx-4-inq-10.1177_00469580251375911 – Supplemental material for Investigating the Acceptability and Feasibility of Three Online Interventions for Caregivers of Infants with Feeding Difficulties [file sj-docx-4-inq-10.1177_00469580251375911.docx]

**Appendix C:**

**Protocol (music intervention)**

**IRAS ID: 296579**

**Version 1.4, 15^th^ April 2021**

**Intervention**

**Duration:** 6 weeks

**Procedure**:

- Participate in 3 groups sessions during Week 1, 2 and 3.
- Careers will engage with Infant Directed Singing (IDS) in moments of infant distress throughout the whole intervention period (Weeks 1-6)

**Measures (before, during and after the intervention)**

1. ***Pre-intervention only***

Firstly, participants will answer a few demographic questions about their home composition, age, infant date of birth, the first three characters of their postcode (to determine socioeconomic status), and their infant feeding method. Given that the engagement with music may be related with past musical experiences and value attributed to IDS, it is important that each carer is administered a musical history questionnaire about their singing habits with the infant **BEFORE** the intervention starts.

The questions pertaining to the pregnancy period are the following (adapted from Persico, 2017):

- Do you sing to your baby when they are unsettled? Yes/No.
- How often did you sing to your baby in the last 48 hours? Not at all/1-2 times a day/3-5 times a day/>5 times a day
- Have you found singing to your unsettled baby helpful in soothing their symptoms? Most of the time/Often/Sometimes/Never
- Did you experience positive feelings whilst singing to your unsettled baby? Most of the time/Often/Sometimes/Never
- Please complete this statement: My baby is content… Most of the time/Often/Sometimes/Never

The next question will be assessed using 5-point Likert-scale response options:

Please complete this statement: When my baby is unsettled, I feel…

1 Useless 2 3 4 5 Able to cope

1 Anxious 2 3 4 5 Calm

1 Guilty 2 3 4 5 Not Guilty

1 Lonely 2 3 4 5 Supported

1 Frustrated 2 3 4 5 Patient

The questions pertaining to the period after birth will also be used to evaluate potential changes in singing behaviours and perceived value of singing from pre- to post-intervention periods (see next section).

1. ***Pre and post-assessment questionnaires***

To examine the effectiveness of the music intervention, the following questionnaires will be administered before and after the 6-week intervention involvement in this study:

Infant feeding method will be assessed using a validated 11-point Likert Scale with percentage response options varying from 100% formula fed to 100% breastfed over the past 48-hour period (Davie, 2018).

*Perceived Maternal Parenting Self-Efficacy (PMPSE) tool (Barnes & Adamson-Macedo, 2007).*

20-item self-report questionnaire to assess perceived parenting self-efficacy with four sub-scales reflecting different parenting domains: care taking procedures, evoking behaviour(s), reading behaviour(s) or signalling, and situational beliefs. Response options include, ‘strongly disagree’, ‘disagree’, ‘agree’ and ‘strongly agree’. Higher scores on this questionnaire reflect higher perceived parenting self-efficacy.

*Edinburgh Postnatal Depression Scale (EPDS; Cox et al., 1987)*

10-item self-report questionnaire administered to screen for depressive symptoms in the postnatal period. It is the most widely used screening scale for postnatal depression. Higher scores indicate higher levels of depression. A clinical cut-off score of ≥13 identifies scores consistent with major depressive disorder, although the self-report measure does not replace a clinical diagnosis.

*Postpartum Specific Anxiety Scale (PSAS; Fallon et al, 2021)*

16-item self-report questionnaire to assess perceived parenting anxiety in the postpartum period. Questionnaire items cover four domains of parenting: psychosocial adjustment to motherhood anxieties, practical infant care anxieties, maternal competence and attachment anxieties, and infant safety and welfare anxieties. Higher scores indicate higher levels of anxiety. Measured using 4 point Likert scale response options from '0 Not at all' to '3 Almost Always'.

*Short Assessment of Patient Satisfaction (SAPS; Hawthorne et al, 2014)*

7-item self-report questionnaire to assess perceived satisfaction with healthcare professional support. Response options include, ‘very satisfied’, ‘satisfied’, ‘Neither satisfied nor dissatisfied’, ‘Dissatisfied’, and, ‘Very dissatisfied’. Higher scores on this scale correspond with greater perceived satisfaction with healthcare professional support.

To evaluate potential changes in singing behaviours and perceived value of singing, the Value of Music Scale (adapted from Cevasco, 2008) will also be used:

*Please rate how much to you agree with each of the following statements using a scale ranging from 0 (completely disagree) to 10 (completely agree)*

- I sing to my baby when she/he is distressed
- I think it is very important to sing to my baby when she/he is distressed
- Singing helps my baby feeling better
- I experience positive feelings whilst singing to my baby

1. ***Weekly measures (Weeks 1-6):***

**DURING** the intervention, in order to quantify engagement with different coping strategies and the state of the infant and the carer we will monitor the following variables on a weekly basis:

- Frequency of engagement with different IDS
  - *How often did you sing to your baby over the past week?*
    - *Every day, most days, about half of the week, rarely, never*
  - *How often did you sing to your baby when she/he was distressed over the past week?*
    - *Always, Usually, About half the times, Rarely, Never*
- Perceived impact on the infant (e.g., amount of crying, general distress, hours of sleep, etc.)
  - *Did you feel that singing to your baby when she/he was distressed helped them feel better.*
    - *Always, Usually, About half the times, Rarely, Never*
- Perceived impact on the carer (e.g., mood, well-being, etc.)
  - *Did you feel that singing to your baby when she/he was distressed helped you cope and feel better? Always, Usually, About half the times, Rarely, Never*

**First group session**

**Duration:** 1h

**Timing:** Start of Week 1

**Aims:**

- To informs carer about the main benefits of Infant Directed Singing (IDS) for the baby and the carer with a particular focus on live singing with songs of kin.
  - IDS can favour self-regulation (including pain), the general promotion of well-being of infant and carers, and the quality of carer-infant interactions (e.g., Haslbeck, 2014; Loewy, 2015). Particularly important for this study is the fact that maternal singing can favour the relaxation and stabilization of the baby, decrease the mother’s anxiety and sense of helplessness, and enable her to participate in the infant’s care and well-being (e.g., Arnon et al., 2014; Filippa et al., 2013).
- To provide carers with information about singing and giving them the confidence to sing to the infants.
  - Modelling of simple musical ideas based on carer/infant vocalisation.
  - Vocalising with physiological cues from infant.
  - Making suggestions and examples of pre-composed songs that may be used with infants, from nursery rhymes to pop songs, tailored to infant development.
  - To ask carers to think about songs of kin (e.g., lullabies) that they would like to use during the intervention. Songs should be soothing (as opposed to exciting or playful). If not possible, suggest possibilities.

**Second group session**

**Duration:** 1h

**Timing:** Start of Week 2

**Aim:**

- To support the carer to sing the song of kin and increase their confidence to do so.
  - Further modelling of how to tailor parent chosen song to infant.
  - Sharing information about what makes a song soothing and why.
  - Allow space for discussion between participants and facilitators, to explore any issues and/or anxieties.

**Third group session**

**Duration:** 1h

**Timing:** Start of Week 3

**Aims:**

- To identify potential problems/difficulties and increase carers’ confidence in singing to their babies.
  - Allow for organic discussion of any issues and/or anxieties.
  - Facilitators to ask more direct questions about obstacles and challenges to infant directed singing.
  - Further modelling and examples given if needed.
  - Allow a space for reflection on aspects participants feel have gone well and share experience and support with each other.

**Focus group.**

**Duration:** 1 hour

**Timing:** After the intervention (start of Week 7)

- Discuss relevant issues related to interventions (e.g., experience, perceived benefits, barriers, etc.)

**References**

Arnon, S., Diamant, C., Bauer, S., Regev, R., Sirota, G., & Litmanovitz, I. (2014). Maternal singing during kangaroo care led to autonomic stability in preterm infants and reduced maternal anxiety. Acta Paediatrica, 103, 1039-1044. doi:10.1111/apa.12744

Cevasco, A. M. (2008). The effects of mothers' singing on full-term and preterm infants and maternal emotional responses. Journal of music therapy, 45(3), 273-306.

Davie, P (2018). Measuring milk: A call for change in quantifying breastfeeding behaviour. *Midwifery, 63,* 6-7

Filippa, M., Devouche, E., Arioni, C., Imberty, M., & Gratier, M. (2013). Live maternal speech and singing have beneficial effects on hospitalized preterm infants. Foundation Acta Pædiatrica, 102(10), 10171020. doi: 10.1111/apa.12356

Haslbeck, F. B. (2014). The interactive potential of creative music therapy with premature infants and their parents: A qualitative analysis. Nordic Journal of Music Therapy, 23(1), 36-70.

Loewy, J. (2015). NICU music therapy: song of kin as critical lullaby in research and practice. Annals of the New York Academy of Sciences, 1337(1), 178-185. doi: 10.1111/nyas.12648

Persico, G., Antolini, L., Vergani, P., Costantini, W., Nardi, M. T., & Bellotti, L. (2017). Maternal singing of lullabies during pregnancy and after birth: Effects on mother–infant bonding and on newborns’ behaviour. Concurrent Cohort Study. Women and Birth, 30(4), e214-e220.
