# Supplementary material for: Investigating the Acceptability and Feasibility of Three Online Interventions for Caregivers of Infants with Feeding Difficulties
Source: Inquiry. 2025 Oct 18;62:00469580251375911. doi: 10.1177/00469580251375911 (PMC12547111; doi:10.1177/00469580251375911)
Supplement: sj-docx-5-inq-10.1177_00469580251375911 – Supplemental material for Investigating the Acceptability and Feasibility of Three Online Interventions for Caregivers of Infants with Feeding Difficulties [file sj-docx-5-inq-10.1177_00469580251375911.docx]

**Appendix D:**

**Protocol (Health education)**

**IRAS ID: 296579**

**Version 1.2, 15^th^ April 2021**

**Intervention**

**Duration:** 6 weeks

**Procedure**:

- Participate in 3 groups sessions (see below) Weeks 1-3
- Ask carers to use skills learnt during group sessions in moment of infant distress weeks 1-6

**Health education-specific measures (before, during and after the intervention)**

***Pre-intervention only***

1. Firstly, participants will answer a few demographic questions about their home composition, age, infant date of birth, the first three characters of their postcode (to determine socioeconomic status), and their infant feeding method. Given that the engagement with health seeking behaviour may be related with past experiences of seeking health information to learn about one’s baby and perceived importance of health information seeking, it would be important that each carer is administered some a short historic questionnaire, eventually focused on their health information seeking habits with the infant **BEFORE** the intervention starts.

- Do you seek health information about your baby’s condition when he/she is unsettled? Yes/No.

- How often did you seek health information about your baby in the last 48 hours? Not at all/1-2 times a day/3-5 times a day/>5 times a day

- Have you found seeking health information about your baby’s condition helpful in soothing your baby’s symptoms when he/she is unsettled? Most of the time/Often/Sometimes/Never

- Did you experience positive feelings whilst seeking health information about your unsettled baby’s condition? Most of the time/Often/Sometimes/Never

- Please complete this statement: My baby is content… Most of the time/Often/Sometimes/Never

The next question will be assessed using 5-point Likert-scale response options:

Please complete this statement: When my baby is unsettled, I feel…

1 Useless 2 3 4 5 Able to cope

1 Anxious 2 3 4 5 Calm

1 Guilty 2 3 4 5 Not Guilty

1 Lonely 2 3 4 5 Supported

1 Frustrated 2 3 4 5 Patient

***Pre- and post-assessment questionnaires***

To examine the effectiveness of the intervention condition, the following questionnaires will be administered before and after the 6-week intervention involvement in this study:

Infant feeding method will be assessed using a validated 11-point Likert Scale with percentage response options varying from 100% formula fed to 100% breastfed over the past 48-hour period (Davie, 2018).

*Perceived Maternal Parenting Self-Efficacy (PMPSE) tool (Barnes & Adamson-Macedo, 2007).*

20-item self-report questionnaire to assess perceived parenting self-efficacy with four sub-scales reflecting different parenting domains: care taking procedures, evoking behaviour(s), reading behaviour(s) or signalling, and situational beliefs. Response options include, ‘strongly disagree’, ‘disagree’, ‘agree’ and ‘strongly agree’. Higher scores on this questionnaire reflect higher perceived parenting self-efficacy.

*Edinburgh Postnatal Depression Scale (EPDS; Cox et al., 1987)*

10-item self-report questionnaire administered to screen for depressive symptoms in the postnatal period. It is the most widely used screening scale for postnatal depression. Higher scores indicate higher levels of depression. A clinical cut-off score of ≥13 identifies scores consistent with major depressive disorder, although the self-report measure does not replace a clinical diagnosis.

*Postpartum Specific Anxiety Scale (PSAS; Fallon et al, 2021)*

16-item self-report questionnaire to assess perceived parenting anxiety in the postpartum period. Questionnaire items cover four domains of parenting: psychosocial adjustment to motherhood anxieties, practical infant care anxieties, maternal competence and attachment anxieties, and infant safety and welfare anxieties. Higher scores indicate higher levels of anxiety. Measured using 4 point Likert scale response options from '0 Not at all' to '3 Almost Always'.

*Short Assessment of Patient Satisfaction (SAPS;* Hawthorne et al, 2014)

7-item self-report questionnaire to assess perceived satisfaction with healthcare professional support. Response options include, ‘very satisfied’, ‘satisfied’, ‘Neither satisfied nor dissatisfied’, ‘Dissatisfied’, and, ‘Very dissatisfied’. Higher scores on this scale correspond with greater perceived satisfaction with healthcare professional support.

*Please rate how much to you agree with each of the following statements using a scale ranging from 0 (completely disagree) to 10 (completely agree)*

- I seek out health information about my baby’s condition
- I think it’s very important to learn about my baby’s condition
- Learning about my baby’s condition helps me to manage them better
- Learning about my baby’s condition helps me to feel positive

***During all intervention weeks (1-6):***

1. **DURING** the intervention, in order to quantify engagement with the intervention and the state of the infant and the carer it would be very important to monitor the following variables on a weekly basis:
   - *How often did you use the techniques learned and advice given over the last week?*
     - *Every day, most days, about half of the week, rarely, never*

Perception of infant’s symptoms that week (e.g., amount of crying, general distress, hours of sleep, etc.

- - *How often did you use the techniques learned and advice given over the last week when he/she was distressed?*
- *Every day, most days, about half of the week, rarely, never*
  - *Did you feel that using the techniques learned and advice given over the last week with your baby when she/he was distressed helped them to feel better?*
- *Every day, most days, about half of the week, rarely, never*
  - *Did you feel that using the techniques learned and advice given over the last week with your baby when she/he was distressed helped you to feel better?*
- *Every day, most days, about half of the week, rarely, never*

1. **AFTER** the intervention, it would be also relevant to measure the perceived effectiveness of treatment as usual. Same measures that were administered before the intervention (1) are to be administered again to assess change over time and effectiveness of the intervention.

**Session 1**

**Duration:** 1 hour (40-minute information provision and 20-minute moderated discussion)

**Timing:** Start of Week 1

**Aims:**

40-minute informational provision portion of the group discussion will focus on reflux, and will cover the following topics:

- What is reflux (what does it mean) and what is normal?
- Physiology (why are babies more prone to reflux)
- What is cow’s milk protein allergy and symptoms?
- General timeline of symptoms (when start, when at its peak, when getting better)

***Management***

- Feeding: volumes, frequency, common problems and solutions
- Medication (pros and cons and how they work)
- When to worry about vomiting, including monitoring output and growth
- Supporting parents, through moderated group discussion (20 minutes) of questions and discussing shared and unique parental experiences and coping strategies.

**Second session**

**Duration:** 1 hour (40-minute information provision and 20-minute moderated discussion)

**Timing:** Start of Week 2

**Aims:**

40-minute informational provision portion of the group discussion, will focus on colic and will cover the following topics:

- What is colic (definition)
- Why does it happen?
- Timeline of symptoms, baby crying graphs – what is normal.
- Coping strategies (ICON, N/A)
- When to worry about crying
- Supporting parents, through moderated group discussion (20 minutes) of questions and discussing shared and unique parental experiences and coping strategies.

**Third session**

**Duration:** 1 hour (40-minute information provision and 20-minute moderated discussion)

**Timing:** Start of Week 3

**Aims:**

40-minute informational provision portion of the group discussion will focus on baby development and weaning, and will cover the following topics:

- Normal development and what to do if you are worried about development
- How to encourage development (tummy time etc)
- Normal sleep patterns including tiredness cues and night time feeding
- Signs of readiness for weaning
- Early weaning pros and cons
- Gagging/ choking (difference between)
- Finger foods v purees
- Should you avoid certain foods in refluxy babies?

**Focus group.**

**Duration:** 1 hour

**Timing:** After the intervention (start of Week 7)

- Discuss relevant issues related to interventions (e.g., experience, perceived benefits, barriers, etc.)

**References**

[Reference removed due to re-identifiable information about the hospital site]

[Reference removed due to re-identifiable information about the hospital site]

Barnes, C. R. & Adamson-Macedo, E. N. (2007). Perceived Maternal Parenting Self-Efficacy (PMP S-E) tool: Development and validation with mothers of hospitalized preterm neonates. JAN Research Methodology, 60(5), 550-561

Cox, J.L., Holden, J.M., Sagovsky, R., 1987. Detection of postnatal depression: development of the 10-item Edinburgh postnatal depression scale. Br. J. Psychiatr. 150 (6), 782–786. https://doi.org/10.1192/bjp.150.6.782

Davie, P (2018). Measuring milk: A call for change in quantifying breastfeeding behaviour. *Midwifery, 63,* 6-7

ICON (N/A). *Parents Advice.* <https://iconcope.org/parentsadvice/>

Taylor, A., Atkins, R., Kumar, R., Adams, D., Glover, V., 2005. A new Mother-to-Infant Bonding Scale: links with early maternal mood. Arch. Wom. Ment. Health 8 (1), 45–51. <https://doi.org/10.1007/s00737-005-0074-z>
