# Supplementary material for: Investigating the Acceptability and Feasibility of Three Online Interventions for Caregivers of Infants with Feeding Difficulties
Source: Inquiry. 2025 Oct 18;62:00469580251375911. doi: 10.1177/00469580251375911 (PMC12547111; doi:10.1177/00469580251375911)
Supplement: sj-docx-6-inq-10.1177_00469580251375911 – Supplemental material for Investigating the Acceptability and Feasibility of Three Online Interventions for Caregivers of Infants with Feeding Difficulties [file sj-docx-6-inq-10.1177_00469580251375911.docx]

**Appendix E: Intervention topic guide questions**

**IRAS ID: 296579**

**Version 1.2, 15 April 2021**

We would like to hear about how you found the intervention. We would like to know why you decided to take part, what it was like for you, and what impact (if any) it has had on you and your baby. We would also like to hear about how things have been since the intervention, if things have improved, and whether you're satisfied with what's happened.

**Accessing the intervention**

What was it that made you decide to sign up to the study?

Were you given enough information to make an informed choice?

Did you have any initial reservations about taking part?

**Experiences of receiving the intervention**

1. **Format and content**
2. **Thinking about when you started taking part in this study, did this happen at a point where you felt that extra support would be helpful for you?**

- Would you have liked to have started taking part earlier? Later?
- Why (not)?

1. **How did you find the sessions overall?**

- Were there any specific sessions you found particularly helpful/unhelpful?
- Was there anything missing that you would have liked?
- How did you find the activities set in between sessions?

1. **How did you find the group nature of the sessions?**

- Anything you particularly liked/disliked about the group environment?

1. **How did you find the online nature of the intervention?**

- Anything you particularly liked/disliked about the online environment?
- How would you feel about participating in the same intervention face to face?
- [For peer support probe both group sessions and WhatsApp]

**II. Delivery**

1. In terms of who led the sessions, what did you think of their approach?

- Anything you found particularly helpful/unhelpful?

1. Was the information they provided helpful?
   - Was there anything less helpful?

**III. Time**

1. How did you find the length of each session?
2. Were the sessions frequent enough/not frequent enough?
3. Were you happy with the number of sessions overall?

**Views on outcomes**

1. How did you find putting the information and skills you learned during the intervention into practice?
2. How do you think that taking part in the intervention has had an effect on you personally (positive/negative)?
   1. Has it helped you?
   2. In what ways?
3. How do you think that taking part in the intervention has had an effect on the way you manage your baby?
   1. Has it helped you?
   2. In what ways?
4. How do you think that taking part in the intervention has had an effect on your baby’s symptoms?
   1. Has it helped them?
   2. In what ways?
5. Did you enjoy the sessions?
6. Are you satisfied with what you were offered?
7. Would you recommend it to other people?
8. Was there anything you didn't like or would change?
