# Supplementary material for: Investigating the Acceptability and Feasibility of Three Online Interventions for Caregivers of Infants with Feeding Difficulties
Source: Inquiry. 2025 Oct 18;62:00469580251375911. doi: 10.1177/00469580251375911 (PMC12547111; doi:10.1177/00469580251375911)
Supplement: sj-docx-7-inq-10.1177_00469580251375911 – Supplemental material for Investigating the Acceptability and Feasibility of Three Online Interventions for Caregivers of Infants with Feeding Difficulties [file sj-docx-7-inq-10.1177_00469580251375911.docx]

**Staff Focus Group Babies in Tune**

**IRAS ID: 296579**

**Version 1.3, 15 April 2021**

I'd like to find out your views about the intervention, why you decided to take part in it, what it was like, and what impact (if any) it has had on you. . I'd also like to find out what's happened since the intervention and whether it has changed the way you work in any way.

**Involvement in the intervention**

What was it that made you decide to work on the study?

Were you provided with sufficient information about the study?

Were you sufficiently involved in the development of the study? Did you have any initial reservations about your role on the project?

**Preparing the sessions**

1. **How did you find preparing the sessions overall?**
   - Were you sufficiently supported?
   - Were there any specific sessions you enjoyed/didn’t enjoy preparing?

**Experiences of delivering the intervention**

1. **Format and content**
2. Thinking about when participants were recruited for this study, did this happen at a point where you felt that the caregivers and infants would find extra support beneficial?

- Should participants be recruited earlier? Later?
- Why (not)?

1. How did you find delivering the sessions overall?
   - Did you find delivering the sessions enjoyable overall?
   - Were there any aspects which were stressful?

- Were there any specific sessions you enjoyed/didn’t enjoy delivering?
- Was there anything missing that you would have liked?
- How did you find the activities set in between sessions?

1. How did you find the group nature of the sessions?

- Anything you particularly liked/disliked about the group environment?

1. How did you find the online nature of the intervention?

- Anything you particularly liked/disliked about the online environment?
- How would you feel about participating in the same intervention face to face?
- [For peer support probe both group sessions and WhatsApp]

III. Time

1. How much time did it take you to prepare the sessions?
2. How did you find the length of each session?
3. Were the sessions frequent enough/not frequent enough?
4. Were you happy with the number of sessions overall?
5. How did being involved in this study impact on your day to day workload?

**Views on outcomes**

1. How do you think that delivering the intervention has had an effect on you personally (positive/negative)?
   1. Has it helped you?
   2. In what ways?
2. How do you think that taking part in the intervention has had an effect on the way families manage their babies?
   1. Has it helped them?
   2. In what ways?
3. How do you think that taking part in the intervention has had an effect on baby’s symptoms?
   1. Has it helped them?
   2. In what ways?
4. Did you enjoy the sessions?
5. Are you satisfied with what you offered?
6. Would you recommend it to other practitioners?
7. Was there anything you didn't like or would change?
