# Supplementary material for: Investigating the Acceptability and Feasibility of Three Online Interventions for Caregivers of Infants with Feeding Difficulties
Source: Inquiry. 2025 Oct 18;62:00469580251375911. doi: 10.1177/00469580251375911 (PMC12547111; doi:10.1177/00469580251375911)
Supplement: sj-docx-8-inq-10.1177_00469580251375911 – Supplemental material for Investigating the Acceptability and Feasibility of Three Online Interventions for Caregivers of Infants with Feeding Difficulties [file sj-docx-8-inq-10.1177_00469580251375911.docx]

**Appendix G:** Supporting evidence with illustrative quotes, conversation analysis on WhatsApp group data.

| **Major Theme** | **Sub-theme** | **Description of theme** | **Example exert** |
| --- | --- | --- | --- |
| Trial-and-error symptom management |  | Comparisons made with infants unafflicted by CMPA, Colic, and GOR[D] intensified one’s distress. Negative interactions with other parents and dismissive clinicians also had adverse effects on maternal mental health. The WhatsApp group was frequently used for sharing infant care practices and non-clinical symptom management techniques for their infants, which aided in normalising the infant's condition. |  |
|  | *Halved problems, shared solutions* | Characterised by difficulties managing infant unsettledness, exacerbated uncertainties about infant development, turn-taking working towards acceptance of infant’s condition, irony, and humour used to prompt candid exchanges. | *Barbara: William went down around half 8.... Woke at half 10, 1am, 3.30am, 5am, 7am and then half 8…Lately he's been waking randomly throughout the night...’*  *Silvia: Awww no is it the 4-month sleep regression? This is all I hear about 😂😂! I put George down at 7 and then dream fed him at 11 and he woke at 4 for a bottle then 6 with little pains he was so windy bless him and then he went back to sleep until 8 for his next bottle! [...]*  *Barbara: Most likely it is... I was reading about that and found a guide as to how to improve sleep at this stage... I use my sleepless nights to read about babies and sleep... Oh the irony 🙈* |
|  |  | Also characterised by frustrations held towards healthcare practitioners e.g., receiving contradictory advice. Sharing one’s unsatisfactory experiences prompted information exchange between group members, as well as recognition and validation of one another through farcifying one’s own experiences and encouraging one another to do what works best for them. | *Silvia: Barbara if you ever want to try neocate I have loads of tins here I can give you […] Although it threw me the other day, the consultant told me not to rule out an allergy […]*  *Barbara: Crazy how little they know about allergies in babies that they would suggest swapping like for like! Thanks Silvia 👍 If there's no improvement in his eczema etc after 2 or 3 weeks on this formula, I'll try the neocate 👍 […] early on, our allocated HV was off sick, [and the replacement] said that if he [baby] didn't have issues with his bowels it couldn't be an allergy... When we eventually got seen at [Hospital]the paediatrician said with his eczema and reflux, it absolutely could be an allergy... [...]*  *Silvia: […] I hope you’ve called the doctor to have that prescription and you’re not paying for it. It's a ridiculous amount of special formulas !! … It sounds like we have had a similar experience of one saying no it’s not an allergy and one saying no it could be! Where does that ever leave us then in knowing for sure […]* |
|  | *Infant care anxieties* | Expressing vulnerability in the peer support group was met with instilled confidence by other group members | *Barbara: The paediatrician actually suggested early weaning for William... I'm afraid to start though because he chokes sometimes when his milk comes up... I'm terrified what would happen if it were food he was bringing up and choking on 😬 xx*  *Silvia: I was going to say I’m considering early weaning! […] It’s scary isn’t it to try but go with your instincts on what you think he can handle and when! It’s hard to make sure we’re not causing any other issues isn’t it xx* |
|  |  | WhatsApp group was used to seek reassurance, other mothers responded drawing on their previous experience to reassure the mother of normal infant behaviour at this developmental stage. Multiparous mother placed by other group members as the ‘knowledgeable, experiences’ advisor. | *Silvia: Does anyone else stress over how much sleep their little one gets in the day 😩🙈 I find myself really stressing over him getting enough daytime sleep because I’m scared it’ll affect the night-time sleep and I just wish I was more chilled out about it […]*  *Madison: […] My little one doesn’t sleep much a day I don’t think but neither did my 7-year-old […] I do feel like some days she sleeps even less if she’s having a bad reflux day x*  *Silvia: Ahh thanks that makes me feel better I just feel like I’m always trying to force him [to sleep] because I see so much about it affecting night sleep and then getting overtired it’s overwhelming when it’s your first and you don’t have anything to compare to 😅 x* |
| ‘Us’ against ‘Them’ |  | The WhatsApp group was often used to promote coping through humour, validation, and normalisation of one another’s experiences. All mothers had received negative judgement and/or insensitive comments from family, healthcare practitioners, and/or other parents, which elicited feelings of shame. The WhatsApp group offered a safe platform for the mothers to keep in perspective that their infant’s condition will improve with time. Finally, the WhatsApp group enabled mothers to share details of infant development that extended past their condition, and to cherish their infant’s progression with their condition. |  |
|  | Frustrated and judged | Expressing empathy for one another’s feelings bridged into sharing one’s own, likened experience of parenting an infant with GOR, reflux and/or CMPA. Comparisons made between their unsettled infant and unafflicted babies resulted in feelings of disappointment. Having space to reflect and share in a candid manner allowed for a more optimistic reflection of one’s circumstances to be maintained | *Madison: […] my poor Dad doesn’t really get to hold her [Billie] much and he’s such a lovely grandad loves just holding them but when they unsettled and crying I know he doesn’t like it so passes her on! […]*  *Silvia: Aww I know the feeling I feel like every time I take George to my mum and ads he cries 🙈 I have 3 nephews and 1 niece all from ages 16 to 11 months and none of them have been like George it’s so sad because I just want him to be happy and content especially when my nephew so close in age is so smiley and happy! […] At least we know we’re not alone! X* |
|  |  | Barbara opened a conversation about her negative experience of attending a parenting group, whereby her infant’s unsettledness was met with intolerance. Again, another group member responded by sharing their personal reservations about attending group sessions, normalising infant behaviour, and reassuring Barbara that she had done the *‘right thing’*. Silvia’s reassuring response prompted Barbara to focus on more positive experiences of attending parenting groups, although worries were still persistent regarding how one appears to other mothers. | *Barbara: (…) I went to a baby group this morning and William brought up milk all over the lovely carpet and screamed any time I tried to put him on his back to join in... When we were leaving I'm sure we were the only ones the group leader did not say "Hope to see you next week" to 🙈 😅 xx*  *Silvia: 😂😂 oh nooo! Do you know that I’ve not taken him to a group yet because I’m so scared of him crying the whole time 🙈 but I just need to do it because babies cry it is what it is! Why should they miss out on anything! Xx*  *Barbara: Absolutely! I take William to a different group every day... We never get through a session without crying but if he enjoys even a small part of it it's worth it 👍 Even though it really is beyond embarrassing when he screams and I can't settle him…Some of the things people say to you are mad though... One mum suggested that I feed him 🙄 Another reassured me that you don't have to stay for the full session 🙄 Would be great if she took her own advice, I thought 😅 xx* |
|  | Feeling like a bad mother | Expressions of emotional vulnerability and insecurity were common conversation triggers. Here, Barbara indicates how her infant’s unsettledness had enveloped her in feelings of shame (blinded for review). As consistently shown during these group interactions, a second mother then intervenes, leading with reassurance and empathy and rationalising maternal difficulties as normal and transient. Rationalising and normalising Barbara’s feelings and experiences served to remove the intensity of their negative valance | *Barbara: I feel like they must just think I'm a rubbish mum! Wish they knew more about it because it's hard to explain xx*  *Silvia: Aww they will not think you’re a rubbish mum at all, as you said they’ve seen it all so they must know how much discomfort he’s in and how difficult it can be to settle him. We will all get there with them being more settled and happy they just need to pass through this unfortunately and for us it will feel like a lifetime but we will look back on it and think how quickly it went I’m sure 🤞🏻☺️ xx*  *Barbara: That's really lovely Silvia 😊 Someday we'll look back at all of this and laugh... Well maybe that's too far... Look back and not cry 🤣 xx* |
|  |  | Avoidance behaviour (a common manifestation of shame; blinded for review) was consequential to receiving insensitive, judgemental comments from other parents. This was met with understanding and validation by the other group members | *Silvia: Oh my god I can’t believe some people honestly, how you can expect not to hear baby’s cry in groups is beyond me 😂 I’m like you though I feel embarrassed if I can’t settle him but I need to just do it! In my head I keep saying I’ll wait until he’s 3 months so is more alert to enjoy it but honestly I think it’s just an excuse in my head xx*  *Barbara: You're absolutely right to wait 3 months. I only did one group for 3 months... [parenting group and baby sensory group] and he used to just scream through it or sleep through it 🙈* |
|  |  | Reassurances from other group members protected the *‘good mother’* (blinded for review) identity from feelings of shame | *Madison: Sorry tough night getting little miss down, her reflux seems to have peaked again! She does still try to spit it out 🙈 I hold her across my legs, so her head is tilted back slightly. It's awful traumatic I think actually for them, but you know what you're doing is to help them isn’t it!! X*  *Barbara: Absolutely! You know you are only doing right by them xx* |
|  | My baby is more than just unsettled | Mothers were keen to attend sessions together, further substantiating their emotional intimacy through face-to-face support. Re-phrasing infant’s unsettledness using humour unified maternal confidence to attend sessions, despite insecurities | *Barbara: … If you're local, you should come along.... We could compete for fussiest baby 🏆*  *[List of days, times, and locations of various baby groups in local area]*  *(…)*  *Silvia: Ahh yeah I am local I’m [area in city] so id definitely come it would be so good to go with someone who doesn’t mind the crying 😅 (…)* |
|  |  | After attending a face-to-face parenting group together, Silvia initiated a conversation by expressing pride in William and George having attended a group session without an unsettled episode. Both mothers acknowledged the non-linear nature of the infant's symptoms. Silvia then goes on to identify feelings of grief regarding the infant's development, which was perceived to be tainted by their condition, which was reaffirmed by Barbara’s views. Both mothers were exhausted with inadequate available support for their managing their infant’s condition-related challenges: | *Silvia: Aww Barbara I’m so proud William and George where the only babies who didn’t cry during the massage 😅 who are these babies 🤣 […]*  *Barbara: The only babies who didn't cry 🤯 Can you believe it? 😅 So proud of both of them 🥰 […]*  *Silvia: Ahh Barbara bless him [George] 🥺😪 it’s so hard isn’t it, the impact it has on enjoying all these moments with them is really sad, and everyone says oh they grow out of it by the time they’re 1! Well my mat leave will be over then and I’ll feel like I’ve not really enjoyed any of it as I could have done if he hadn’t had this 🥺 hope he has a better day today 🤞🏻🤞🏻 xx*  *Barbara: You couldn't have put it better Silvia... I feel exactly the same 🥺 I don't want to wish the first year of my baby's life away just to see the back of something that there should be help for! Xx* |
