# Supplementary material for: Investigating the Acceptability and Feasibility of Three Online Interventions for Caregivers of Infants with Feeding Difficulties
Source: Inquiry. 2025 Oct 18;62:00469580251375911. doi: 10.1177/00469580251375911 (PMC12547111; doi:10.1177/00469580251375911)
Supplement: sj-docx-9-inq-10.1177_00469580251375911 – Supplemental material for Investigating the Acceptability and Feasibility of Three Online Interventions for Caregivers of Infants with Feeding Difficulties [file sj-docx-9-inq-10.1177_00469580251375911.docx]

Appendix H: Thematic analysis supporting illustrative quotes

| Major theme | Sub-theme | Example quote | Interpretation |
| --- | --- | --- | --- |
| Effective design and delivery | A personal touch | *“...getting a personal phone call to say this study might suit you and your baby was quite nice. And actually, I signed up probably for that reason and I might not have had [it] been generic in the hospital.” (Barbara, peer support).*  *“I think the delivery was really good…having the kind of balance of there being information on the slides…while also having the balance of being able to interact with each other, and kind of know our thoughts and, and what would be useful for us and things like that.” (Silvia, peer support).* | The personalised nature of recruitment was commended: having direct interactions with delivering members of the research team played a pivotal role in motivating participants to commit time to the intervention. |
|  |  | *“I'm not sure if there were ten parents, I'm not sure you'd have the kind of closeness in the group that you have, because on a message, when you're only one of three and a message comes up, there's sort of an onus on you to respond, and you're, you are very visible in the group.” (Barbara, peer support).* | The peer support arm was intended for ten participants, which was not achieved due to exceptional recruitment difficulties. However, value was placed on the small-group nature of the WhatsApp group. This was attributed to increased feelings of personal responsibility and social cohesion when in a smaller group. |
|  |  | *“It was a good blend I thought of clinical practice, art practice and research practice. I think that’s definitely one of the strengths and probably one of the most slightly innovative aspects of the project and to bring all those together.” (XX, staff).* | For the research team, multidisciplinary collaboration and co-design was critical for impactful research. Contributing diverse expertise and life experiences enhanced study benefit. |
|  | Accessibility and instilled confidence | *“If it were face-to-face every week, that would be far too much … I think you would find it difficult to for people to sign up to that, because it's a lot and it's a lot to work everyone's schedule and things like that, and even just getting your baby out of the house at that time can be hard.”* *(Babara, peer support)*. | Online delivery was thought to improve intervention accessibility, especially for parents who were struggling to manage a busy schedule. |
|  |  | *“If it [WhatsApp group] had gone quiet for a couple of days, you [XX] kind of just put something into a general message to ask how everyone was doing. And I think that was enough to then get the conversation going again…it was just the right amount of inputs really… I’ve got the most value out of the WhatsApp group.” (Silvia, peer support).* | The WhatsApp group widely benefited participants in terms of normalising infantile symptoms. Facilitative prompts used were perceived to be appropriate. |
|  |  | *“...because I know other babies that are like this now... that’s had a huge impact emotionally, definitely, yeah. There’s less self-blame, less negative emotions around it, the emotions around it now are much more positive*.” *(Barbara, peer support).*  *“Even knowing that there’s other babies in that situation makes you attribute less of it to yourself, and actually, conversationally, because you’re talking to other moms, I’ve tried this, I’ve tried this, you’re not in the conversation. So I suppose you’re not the problem”. (Barbara, peer support).* | Engaging with other parents with shared life experiences ascribed benefits to maternal wellbeing and confidence. |
|  |  | *“I think the most I got out of it for me as well is realizing that I needed to take a little bit of time for myself. I think going through that kind of session where there was kinds of things that we need to do for ourselves as well to be able to have a little break, like mentally. And I think that I have definitely started putting things in place after that just to know that I needed a break from [baby]...”* *(Silvia, peer support).*  *“I get up before [baby], I now do that, after we talked about that [in the group session], I now do that religiously… and actually take that time to myself in the morning to get ready and have a coffee and have all that before I get him, before he wakes.” (Barbara, peer support).* | Session three of the peer support arm, which focused on self-care and compassion, was particularly valued by participants for fostering emotional resilience. |
| Study weaknesses | Delayed point of entry | *“By the time you come to [Hospital], there's a couple of things that have to happen…you’ll have maybe spoken with a health visitor, you might have gone to [emergency department] ...[Hospital] is sort of a last resort, really.” (Barbara, peer support).*  *“...we were too late in the intervention, by the time they [parents] have gone to visit their health visitors, GPs, end of the line was [Hospital] ...they would have wanted [the intervention] when [their infant was] two months old not when they were four months old.” (XX, Staff)* | Participants noted that study retention may have been improved by recruiting at an earlier point in their infant’s illness trajectory. Staff accounts were congruent with maternal concerns. |
|  |  | *“… [it would be good if] in that initial meeting with the health visitor or your midwife, for you to be invited to the study then, because actually with things like this they tend to be at their worst at the very beginning and then they get easier as time goes on.”* *(Barbara, peer support).* | By recruiting earlier, for example through universal health visiting services, eligible caregivers could be approached when level of need was more appropriate. |
|  |  | *“…speak to health visitors and give them the information, because I think there's a lot of people who kind of just accept that the baby might have colic,and might not necessarily take the baby to kind of follow up anything” (Silvia, peer support).* | Some participants were concerned that recruitment delays might have inadvertently resulted in some parents going without much needed support. |
|  | A mis-match of the formal and informal | *“…what [the intervention] boils down to is Zoom and a WhatsApp group and it's nothing more complicated than that. So just to have that summarized in a sheet I think will be really helpful.” (Barbara, peer support).*  *“I think the information sheet was terrible. I don’t think I’ve ever managed to read through it all.” (XX, Staff).* | Participant-facing documentation was described as being overly convoluted. Recommendations were made to include a leaflet which clearly summarised participant responsibilities prior to study screen-in. |
|  |  | *“I don't think the information sheet really outlined exactly what was like expected of us really, or what we were like, what was going to happen. And then she [recruiting consultant] didn't really know much about it herself to kind of tell me about it really either.”* *(Silvia, peer support).* | Some recruiting members of staff at [Hospital] lacked study insight, which further added to caregiver confusion when deciding whether to take part in the intervention: |
|  |  | *“I think how you’re called to the study doesn't reflect actually the running of the study. The running of the study is very informal, very relaxed, very in fact, it's to promote openness and engagement and it works around you and your baby…how you're actually called to the study just doesn't marry up with actually that’s how the study runs.”* *(Barbara, peer support).* | The clinical environment, from which parents were recruited, contrasted with the informal nature of the intervention. This resulted in ill-reflective study expectations on screening into the study. |
|  |  | *“[Hospital is a], very professional, clinical, formal environment. So, you feel like, what's being asked of you is also professional clinical formal ...you tend to have quite a close, friendship, informal sort of relationship with your health visitor. So, your health visitor relaying that study to you, I think you're more likely to take it up.” (Barbara, peer support).* | Recruiting in the community was perceived to be a more appropriate fit for the nature of the current study. |
|  |  | *“...Some [participants] might not want music, they might have wanted peer support, or health education. But the nature of the design and the amount of referrals you were getting at [hospital] we couldn’t have randomised or given them any kind of choice in the matter.” (XX, Staff).* | Staff believed allowing participants to select their intervention arm would further encourage participant retention. |
|  | Hybrid delivery and condensed sessions | *“…put an extra session in where you do meet face to face just for an informal chat, really…it could help break the ice a little bit.”* *(Silvia, peer support).*  *“By the time it actually came about to start running [the intervention] everyone was sick to death of Zoom.” (XX, Staff)* | Although online session delivery was preferred among participants, introducing an *‘initial f*ace-to-face session was suggested, to facilitate initial rapport-building. Offering a hybrid delivery would also combat screen fatigue. |
|  |  | *“if your baby is napping, then 45 minutes to an hour is, your baby will start to wake up after that …if [the Zoom session] could be condensed to 45 minutes, that would be great”.* *(Barbara, peer support).* | Condensing sessions to 45-minutes was noted as being more suitable for fitting around childcare commitments. |
|  |  | *“[What if] people needed something immediate, no? Is the format actually a page where they can get all the information, have the information, videos, demonstrate you know that they can immediately go home and engage with that and have all the support in a webpage.” (XX, Staff).*  *“For peer support you could have forums online with moderators, maybe even channels dedicated to first time mothers, second time mothers. Places where people can congregate [between intervention sessions].” (XX, Staff).* | Recommendations were made to develop online resources for use outside of scheduled sessions. This would further extend intervention benefit beyond the lifespan of the project. |
